# Human carnitine biosynthesis proceeds *via* (2*S*,3*S*)-3-hydroxy-*N* ^*ε*^-trimethyllysine†
†Electronic supplementary information (ESI) available: Synthesis procedures, assay conditions, NMR assignments and spectra, and MS analyses. See DOI: 10.1039/c6cc08381a



**DOI:** 10.1039/c6cc08381a

**Published:** 2016-11-29

**Authors:** Robert K. Leśniak, Suzana Markolovic, Kaspars Tars, Christopher J. Schofield

**Affiliations:** a Department of Chemistry, University of Oxford, Chemistry Research Laboratory, 12 Mansfield Road, Oxford OX1 3TA, UK. Email: christopher.schofield@chem.ox.ac.uk; b Biomedical Research and Study Centre, Ratsupites 1, LV1067 Riga, Latvia

## Abstract

*N* ^*ε*^-Trimethyllysine hydroxylase (TMLH) catalyses the first step in mammalian biosynthesis of carnitine, which plays a crucial role in fatty acid metabolism. The stereochemistry of the 3-hydroxy-*N* ^*ε*^-trimethyllysine product of TMLH has not been defined. We report enzymatic and asymmetric synthetic studies, which define the product of TMLH catalysis as (2*S*,3*S*)-3-hydroxy-*N* ^*ε*^-trimethyllysine.

## Abstract 2

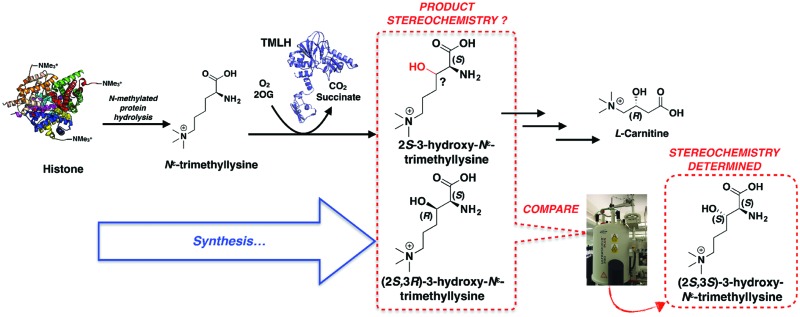
The stereochemistry of human trimethyllysine hydroxylase was determined to be (2*S*,3*S*)-3-hydroxy-*N* ^*ε*^-trimethyllysine by comparison to asymmetrically synthesised (2*S*,3*R*)-3-hydroxy-*N* ^*ε*^-trimethyllysine.

## 

Carnitine plays key roles in mammalian metabolism by enabling the transport of fatty acids into mitochondria as *O*-acyl carnitine esters and in maintaining acetyl group homeostasis.^1–3^ There is considerable biomedical interest in carnitine and its biosynthesis. Carnitine is biosynthesised from (2*S*)-*N* ^*ε*^-trimethyllysine (TML, (**1**)),^4^ which is derived from naturally-occurring TML residues in proteins following proteolysis.^5–7^ Two 2-oxoglutarate (2OG)-dependent oxygenases, *N* ^*ε*^-trimethyllysine hydroxylase (TMLH) and γ-butyrobetaine hydroxylase (BBOX), catalyse the first and final steps of carnitine biosynthesis, respectively (Fig. 1).^8,9^ BBOX is one of the proposed targets of Meldonium (Mildronate, THP, Met-88),^10,11^ a drug that is used for treatment of cardiovascular disease^12^ and by athletes due to perceived performance-enhancing properties.^13,14^ Carnitine is proposed to promote atherosclerosis by acting as a precursor for trimethylamine oxide.^15^ There are also reported links between TMLHE gene mutations and autism in males.^16–18^ Whilst BBOX has been extensively characterised, including by detailed kinetic and biophysical studies,^19,20^ relatively little is reported on TMLH,^21^ notably including on the stereochemistry of the product of its catalysis.

**Fig. 1:**
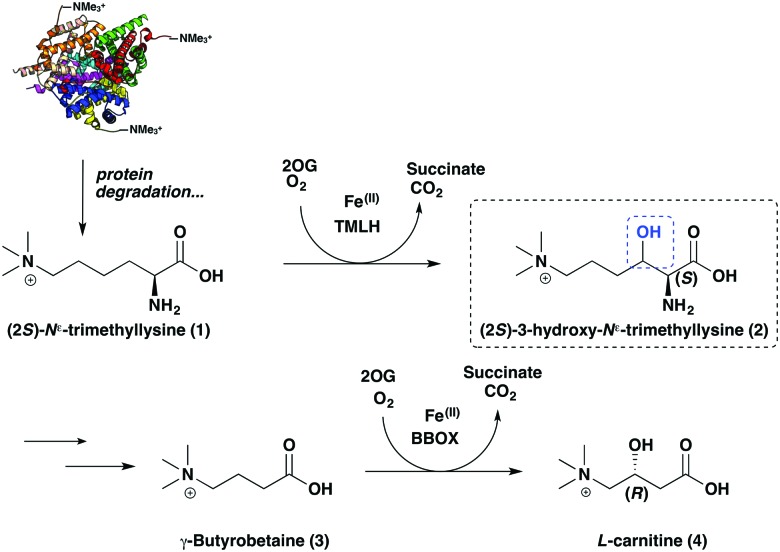
Carnitine biosynthesis. The first and final steps of l-carnitine biosynthesis in mammals are catalysed by the 2-oxoglutarate-dependent oxygenases, *N* ^*ε*^-trimethyllysine hydroxylase (TMLH) and γ-butyrobetaine hydroxylase (BBOX).

To define the stereochemistry of the TMLH-catalysed 3-hydroxy-*N* ^*ε*^-trimethyllysine (3HO-TML) product (**2**), we investigated the asymmetric synthesis of (2*S*,3*R*)-3HO-TML (**13**) (Scheme 1). We employed the Dixon methodology,^22^ which involves Ag(i)-catalysed aldol-type reactions in the presence of a cinchona alkaloid-derived pre-catalyst (**7**).^22,23^ We envisaged this approach could enable the requisite introduction of differently protected *N* ^*α*^- and *N* ^*ε*^-amino groups in a precursor of (**13**). Thus, dibenzyl aldehyde (**6**), prepared in two steps from (**5**), was reacted with *tert*-butyl isocyanoacetate in the presence of Ag_2_O and the pre-catalyst (**7**) to yield *trans*-oxazoline (**8**) (*J*_2–3_ = 7.0 Hz)^22,24^ in good yield (78%; Scheme 1).

**Scheme 1:**
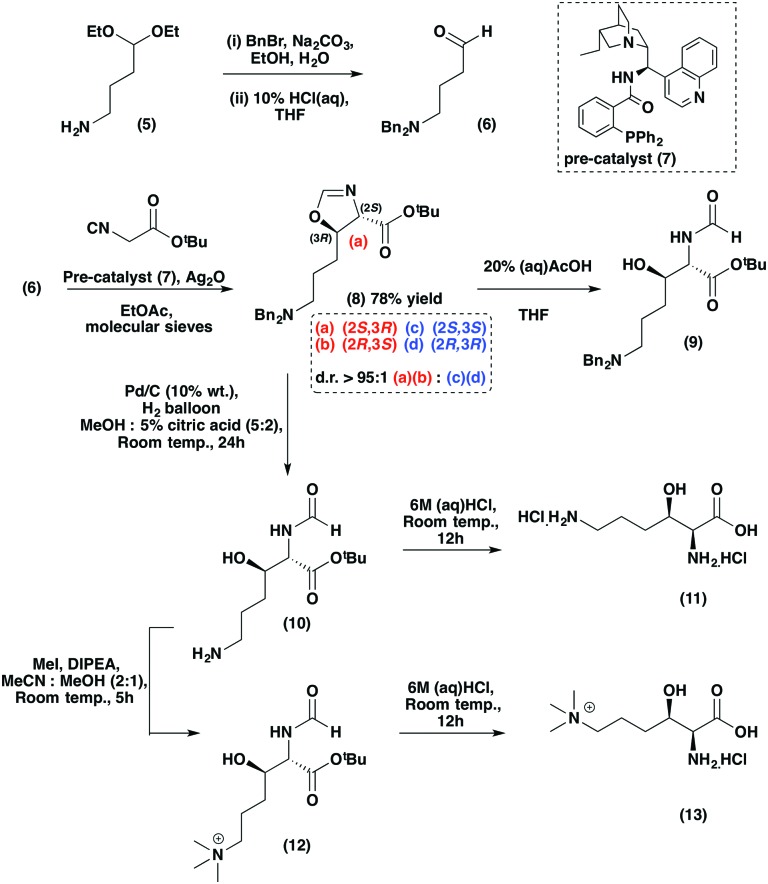
Stereoselective synthesis of (2*S*,3*R*)-3-hydroxy-*N* ^*ε*^-trimethyllysine (**13**) and (2*S*,3*R*)-3-hydroxylysine (**11**) *via* oxazoline (**8**). The oxazoline ring hydrolysis and reduction steps can be carried out separately or *via* a one-pot reaction, as displayed.

Importantly, high diastereoselectivity favouring the *trans*-diastereomers (2*S*,3*R*/2*R*,3*S* : 2*S*,3*S*/2*R*,3*R*; d.r. > 95 : 1) of (**8**) was observed (2*S*,3*R* : 2*R*,3*S*; 3 : 1 inferred from analyses on (**9**)). Oxazoline (**8**) was unstable at room temperature (and over prolonged periods at –20 °C), decomposing to give formamide (**9**). We found that conversion of (**8**) to (**9**) is promoted by aqueous citric acid, as reported for other oxazolines,^22^ or by aqueous acetic acid in THF in near quantitative yield (Scheme 1). The stereochemistry of the major stereoisomer of formamide (**9**) was assigned as (2*S*,3*R*) by ^1^H NMR analysis of the corresponding Mosher's esters (Fig. S1, ESI†).^25,26^

(2*S*,3*R*)-3-Hydroxylysine (3HO-Lys) (**11**) was efficiently obtained from (**8**) using H_2_/Pd/C in aqueous citric acid followed by the removal of formamide and *tert*-butyl ester protecting groups *via* acid hydrolysis to give (**11**) (Scheme 1). The reduction and hydrolysis steps to give (**10**) from (**8**) *via* (**9**) were initially carried out separately; however, use of MeOH/5% citric acid as a solvent during hydrogenation enabled one-pot conversion of (**8**) to (**10**) in high yield (96%). Comparison of the optical rotation of 3HO-Lys (**11**) with that of enantiopure (2*S*,3*R*)-3HO-TML^24,27^ implied an e.r. for (**11**) as ∼3 : 1, consistent with the stereoselectivity observed during oxazoline (**8**) formation.

3HO-TML (**13**) was then synthesised from (**8**) *via* initial hydrogenation in acidic solvent to give the *N* ^*ε*^-amine (**10**). Following treatment with methyl iodide to give (**12**), acid promoted hydrolysis yielded (**13**) with an e.r. of 3 : 1 in favour of (2*S*,3*R*)-3HO-TML (Scheme 1, (**13**)), as determined by Mosher's analysis of the corresponding amide (Fig. S2, ESI†).

We then investigated the stereochemistry of the TMLH-catalysed product (**2**) using recombinant TMLH^28^ and synthetic (2*S*,3*R*)-3HO-TML (**13**) as a standard for comparison by NMR and amino acid analysis. (2*S*)-*N* ^*ε*^-Trimethyllysine (**1**) was converted to 3-hydroxy-*N* ^*ε*^-trimethyllysine (**2**), as shown by 1D and 2D NMR analyses (Fig. 2A and Fig. S3A–F, ESI†). Addition of the synthetic (2*S*,3*R*)-3HO-TML standard (**13**) to the TMLH reaction mixture led to the appearance of non-redundant peaks, implying the TMLH-catalysed product to be the (2*S*,3*S*)-stereoisomer (**14**) (assuming retention of the (2*S*)-stereochemistry in the TML substrate; Fig. 2C and Fig. S3C, ESI†). This assignment was validated by amino acid analysis, using derivatisation with 6-aminoquinolyl-*N*-hydroxysuccinimidyl carbamate. The enzyme-catalysed product and synthetic standard have identical masses (observed *m*/*z* = 375.2114), but were clearly separated by ultra performance liquid chromatography (UPLC). These results reveal exclusive (>95%) formation of the (2*S*,3*S*)-stereoisomer (**14**) as the TMLH-catalysed product (Fig. 2B and Fig. S4, ESI†).

**Fig. 2:**
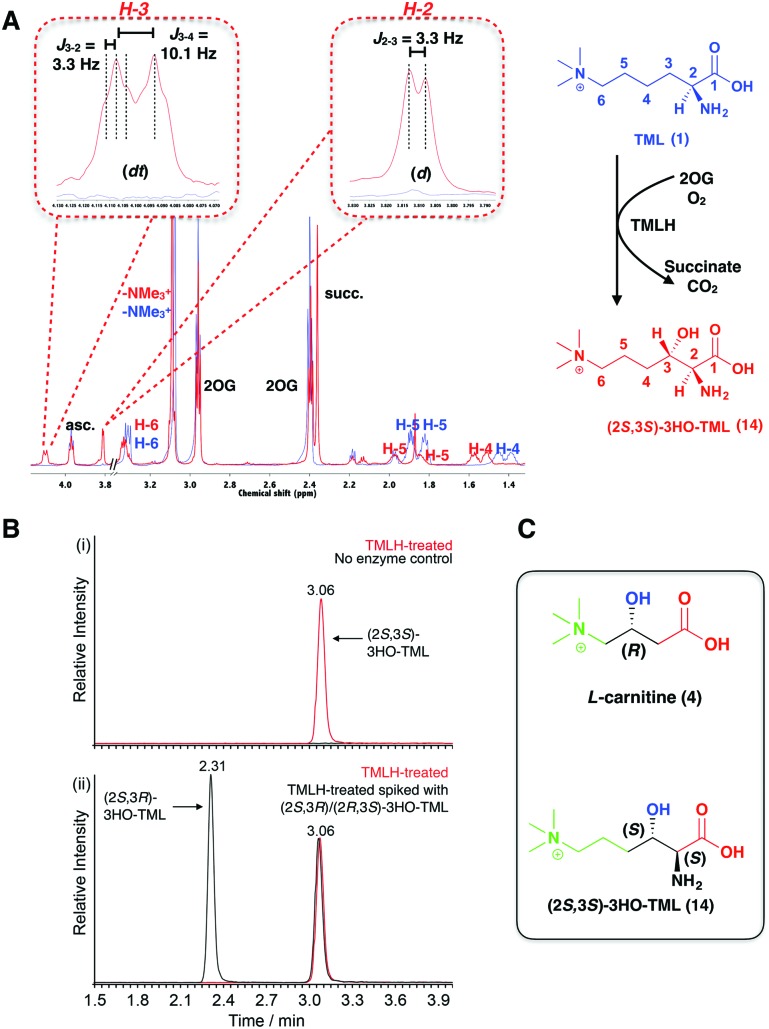
TMLH catalysis produces (2*S*,3*S*)-3-hydroxy-*N* ^*ε*^-trimethyllysine (**14**). (A) ^1^H NMR assignment of the product resulting from TMLH-catalysed C-3 hydroxylation of (2*S*)-*N* ^*ε*^-trimethyllysine (**1**). Superimposition of ^1^H NMR spectra of the reaction mixture before (blue) and after (red) addition of TMLH shows 3HO-TML formation. Signals arising between *δ* = 3.5–3.75 ppm (including glycerol) are omitted for clarity. (B) Overlaid extracted ion chromatograms (*m*/*z* = 375.2, corresponding to the mass of derivatised 3HO-TML) for (i) TML incubated with (red) or without (black; at baseline) TMLH and (ii) TMLH-treated TML (red) and TMLH-treated TML spiked with synthetic (2*S*,3*R*)-3HO-TML ((**13**), black). (C) The stereochemistry of TMLH- and BBOX-catalysed hydroxylation is the same relative to the quaternary ammonium and carboxylate groups.

The overall results define the stereochemical outcome of the TMLH-catalysed hydroxylation of TML as (2*S*,3*S*)-3-hydroxy-*N* ^*ε*^-trimethyllysine (**14**). Interestingly, BBOX catalyses hydroxylation of γ-butyrobetaine (**3**) to give carnitine (**4**) with the (3*R*)-stereochemistry (Fig. 1). Thus, the stereochemical outcomes of TMLH and BBOX catalysis are the same relative to the trimethylammonium and carboxylic acid groups (Fig. 2C), reflecting the likely common evolutionary origins of TMLH and BBOX, as revealed by structural analyses.^19,29,30^

The results also highlight the continued important role of synthesis, including *via* efficient asymmetric catalysis, for biomolecular structural assignments. Modern proteomic and other mass spectrometry (MS) methodologies are identifying many new potential post-translational modifications (*e.g.* JMJD4-catalysed formation of C-4 hydroxylysine),^31,32^ the regio- and stereochemistries of which need to be confirmed, *e.g.* by NMR, high resolution MS analyses and, at least in our view, wherever possible by comparison with synthetic standards.

We thank the Wellcome Trust, Biotechnology and Biological Sciences Research Council, the British Heart Foundation (R. K. L.), and the Clarendon Fund (S. M.) for funding. We thank Dr Jürgen Brem, Dr Michael A. McDonough, and Dr Sarah E. Wilkins for helpful advice.
