# Supplementary material for: Human carnitine biosynthesis proceeds via (2S,3S)-3-hydroxy-N ε-trimethyllysine
Source: Chem Commun (Camb). 2016 Nov 29;53(2):440–2. doi: 10.1039/c6cc08381a (PMC5644716; doi:10.1039/c6cc08381a)
Supplement: Supplementary file 1 [file CC-053-C6CC08381A-s001.pdf]

Electronic Supplementary Material (ESI) for Chemical Communications.
This journal is © The Royal Society of Chemistry 2016

Supporting Information:

Human carnitine biosynthesis proceeds via (2*S*,3*S*)-3-hydroxy-N^{ε}-trimethyllysine

Robert K. Leśniak[a], Suzana Markolovic[a], Kaspars Tars[b], and Christopher J. Schofield[a]*

[a] *Department of Chemistry, University of Oxford, Chemistry Research Laboratory, 12 Mansfield Road, Oxford OX1 3TA, United Kingdom.*

[b] *Biomedical Research and Study Centre, Ratsupites 1, LV1067 Riga, Latvia.*

*To whom correspondence should be addressed. Email: christopher.schofield@chem.ox.ac.uk. Telephone: +44 (0)1865 285 000. Fax: +44 (0)1865 285 002. Address: Chemistry Research Laboratory, 12 Mansfield Road, Oxford, OX1 3TA, United Kingdom.

Contents

General Experimental Methods ... 3
Enzyme Production ... 3
NMR materials and methods ... 3
Amino acid analysis ... 4

Figures ... 5
Figure S1A ^{1}H NMR spectrum of the Mosher's esters resulting from the reaction of formamide (9) with (*S*)-Mosher's acid ... 5
Figure S1B ^{1}H NMR spectrum of the Mosher's esters resulting from the reaction of formamide (9) with (*R*)-Mosher's acid.. ... 5
Figure S1C ^{1}H NMR Mosher ester analysis of the C-3 alcohol of formamide (9) ... 6
Figure S1D ^{19}F NMR Mosher ester analysis of the C-3 alcohol of formamide (9) ... 7
Figure S2 ^{1}H NMR spectrum of the crude aqueous reaction mixture of quaternary amine (13) and (*R*)-Mosher's acid chloride. ... 8
Figure S3A ^{1}H-^{1}H COSY spectrum of TMLH-catalysed TML hydroxylation. ... 9
Figure S3B ^{1}H-^{13}C HSQC spectrum of TMLH-catalysed TML hydroxylation ... 10
Figure S3C ^{1}H NMR spectrum of TMLH-catalysed (2*S*,3*S*)-3HO-TML (14) spiked with synthetic (2*S*,3*R*)/(2*R*,3*S*)-3HO-TML (13) post-HPLC purification. ... 11
Figure S3D ^{1}H NMR spectra of TMLH-catalysed TML hydroxylation is coupled to 2OG conversion to succinate monitored over time (δ = 2.05 - 3.20 ppm). ... 12
Figure S3E ^{1}H NMR spectra of TMLH-catalysed TML hydroxylation monitored over time (δ = 2.95 - 3.95 ppm). ... 13
Figure S3F Numbering used in TMLH product assignment. ... 13
Figure S4 Amino acid analysis of TML-catalysed hydroxylation product. ... 14
Figure S5A ^{1}H NMR spectrum of (2*S*,3*R*)/(2*R*,3*S*)-3HO-Lys (11) ... 15
Figure S5B ^{13}C NMR spectrum of (2*S*,3*R*)/(2*R*,3*S*)-3HO-Lys (11) ... 15
Figure S6A ^{1}H NMR spectrum of (2*S*,3*R*)/(2*R*,3*S*)-3HO-Lys (11) ... 16
Figure S6B ^{13}C NMR spectrum of (2*S*,3*R*)/(2*R*,3*S*)-3HO-Lys (11) ... 16
Figure S7A ^{1}H NMR spectrum of (2*S*,3*R*)/(2*R*,3*S*)-3HO-TML (13). ... 17
Figure S7B ^{13}C NMR spectrum of (2*S*,3*R*)/(2*R*,3*S*)-3HO-TML (13). ... 17

Synthesis ... 18
General Considerations ... 18
Dibenzyl-4,4-diethoxybutan-1-amine ... 19
4-(dibenzylamino)butanal (6) ... 20
N-[(9*R*)-10,11-dihydrocinchonan-9-yl]-2-(diphenylphosphino)benzamide (7)[3] ... 20
tert-Butyl (2*S*,3*R*)-3-(6-(dibenzylamino)propyl)-2,3-dihydrooxazole-2-carboxylate (8) ... 22
tert-Butyl (2*S*,3*R*)-6-(dibenzylamino)-2-formamido-3-hydroxyhexanoate (9) ... 23
tert-Butyl (2*S*,3*R*)-6-amino-2-formamido-3-hydroxyhexanoate (10) ... 24
(2*S*,3*R*)-2-amino-2-carboxy-3-hydroxy-*N*,*N*,*N*-trimethylpentan-6-aminium (13) ... 25
(2*S*,3*R*)-2,6-Diamino-3-hydroxyhexanoic acid di-hydrochloride salt (11) ... 26
Synthesis of Mosher's esters for stereochemical investigation of (9) ... 27
Synthesis of Mosher's amides of (13) ... 27

References ... 28

General Experimental Methods

Enzyme Production

Purified recombinant TMLH-MBP (MBP, maltose binding fusion protein) protein was prepared at the Latvian Biomedical Research and Study Centre (Riga, Latvia);[1] the MBP tag was cleaved as described. TMLH-MBP was incubated with the rTEV protease (20:1 TMLH-MBP:rTEV; a kind gift from Dr. Illaria Pettinati, University of Oxford) in 20 mM Tris, 200 mM NaCl, pH 7.5 overnight at 4°C. Following the protease-catalysed cleavage reaction, the resultant solution was incubated with dithiothreitol (1mM final concentration) overnight at 4°C, then purified by gel-filtration using a 16/600 Superdex S200 120ml HiLoad column (GE Healthcare Life Sciences) attached to an AKTA FPLC system; the protein was eluted in 50 mM Tris, 200 mM NaCl, pH 7.5.

NMR materials and methods

NMR experiments were carried out using a Bruker AVIII 700 MHz instrument equipped with a 5mm inverse TCI cryoprobe and 3mm Bruker Match tubes. The single-pulse nutation method was used to calibrate pulses (Bruker pulsecal routine). Tris-d_{11} and D_2O were obtained from Sigma-Aldrich.

TMLH assay incubations for NMR analysis comprised: 500 µM N^{ε}-trimethyllysine hydrochloride, 1500 µM (final concentration) 2-oxoglutarate (2OG, monosodium salt), 500 µM *L*-ascorbate (disodium salt), 150 µM ammonium iron(II) sulfate hexahydrate ($Fe^{(II)}$), 10 µM TMLH, and 50 mM Tris-d_{11}, 200 mM NaCl, pH 8.0 buffer. The $Fe^{(II)}$ solution was freshly prepared before each reaction by diluting a 100 mM $Fe^{(II)}$ solution stock in 20 mM HCl with D_2O, as required, prior to addition to the reaction mixture. Reagent stock solutions were made up in D_2O immediately prior to use; the enzyme stock solution was made up in 50 mM Tris-d_{11} with 200 mM NaCl by buffer exchanging the original enzyme solution (50 mM Tris, 200 mM NaCl, pH 7.5). Reactions were initiated by addition of the $Fe^{(II)}$ solution. The first NMR spectrum was measured 240 sec after the start of reaction. All ^{1}H spectra were obtained using 64 scans. Control reactions were recorded without enzyme and metal.

2D NMR experiments used to assign the TMLH-catalysed turnover product were recorded after no further conversion of TML to 3HO-TML or 2OG to succinate was observed by ^{1}H NMR. Initial reactions were performed in Tris-d_{11} pH 8.0, although pH 7.5 was determined to be optimal. All subsequent reactions were performed at pH 7.5 unless otherwise stated. 2D experiments used: ^{1}H-^{1}H COSY and multiplicity edited ^{1}H-^{13}C HSQC NMR. Synthetic standards were characterised using a Bruker AVIII HD 400 instrument and 5 mm tubes.

Amino acid analysis

Samples were prepared as described for NMR analyses and subsequently freeze-dried. Freeze-dried samples were resuspended in borate buffer pH 9.0 and derivatised by 6-aminoquinolyl-*N*-hydroxysuccinimidyl carbamate (AQC) in acetonitrile at 25°C according to the AccQ-Tag™ Ultra Derivatisation Protocol (Waters, USA). LC-MS analyses were performed as described[2] using a Waters Acquity ultra performance liquid chromatography system coupled to a Xevo® G2-S QTof mass spectrometer equipped with an electrospray ionisation source (Waters, USA). Gradient conditions for separation were carried out as described in the AccQ-Tag™ Ultra Derivatisation Protocol (Waters, USA).

Conditions for positive ion mode ESI-MS detection were as follows: desolvation temperature, 600°C; source temperature, 100°C, capillary voltage, 3000 V; sample cone voltage, 20 V; cone gas flow, 30 L/min; and desolvation gas flow 1000 L/min. MS data were acquired and extracted ion chromatograms were produced for m/z values of either 359.2 or 375.2, corresponding to the theoretical masses of AQC-derivatized N^{ε}-trimethyllysine (359.2083) or hydroxy-N^{ε}-trimethyllysine (375.2032), respectively. Extracted ion chromatograms were smoothed to the mean (number of smooths, 2; smooth window channels, 3), and total ion current chromatograms were further baseline subtracted (polynomial order, 1; below curve %, 40; tolerance, 0.01).

Figures

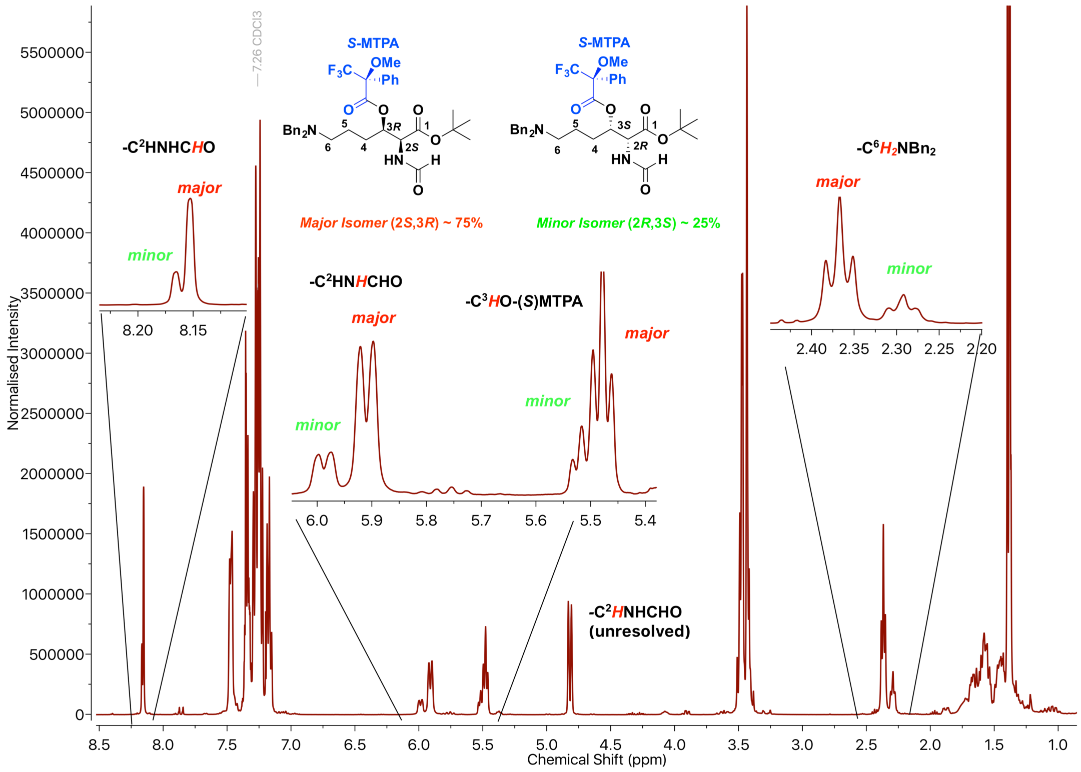

Figure S1A ^{1}H NMR spectrum of the Mosher's esters resulting from the reaction of formamide (9) with (*S*)-Mosher's acid. The signals used in the stereochemical assignment are enlarged. As yet, separation of the mixture of stereoisomers has been unsuccessful by HPLC or crystallisation, hence the Mosher's method was used.

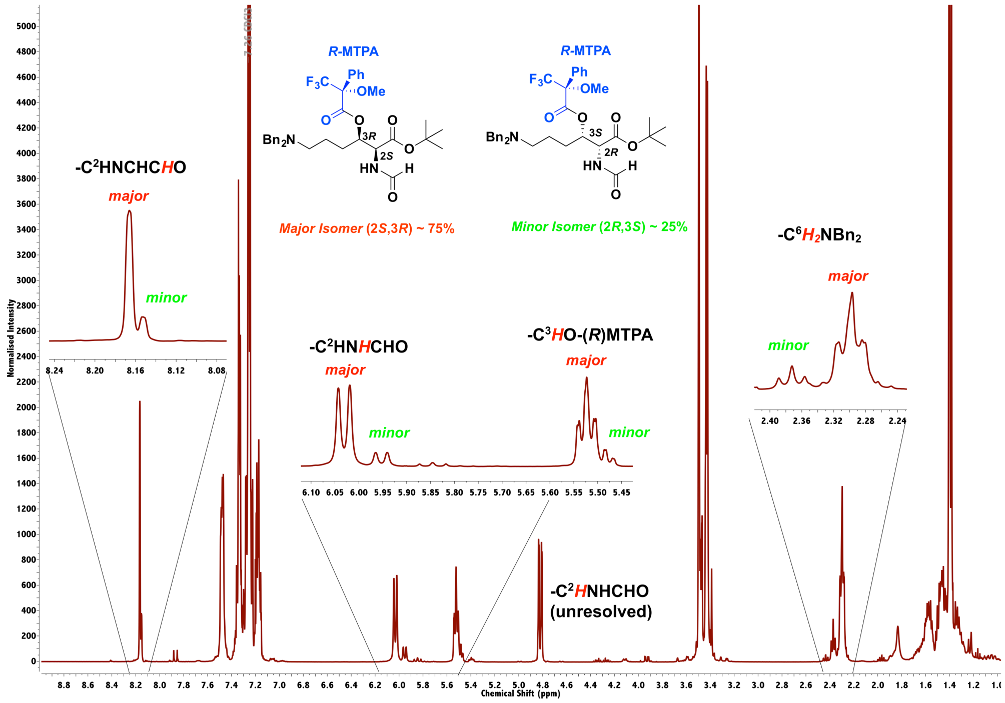

Figure S1B ^{1}H NMR spectrum of the Mosher's esters resulting from the reaction of formamide (9) with (*R*)-Mosher's acid. The signals used in the stereochemical assignment are enlarged.

(9)-[(*S*)-MTPA] **(9)-[(*R*)-MTPA]**

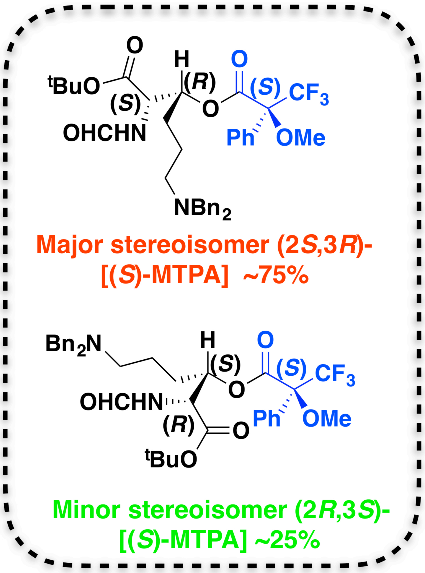

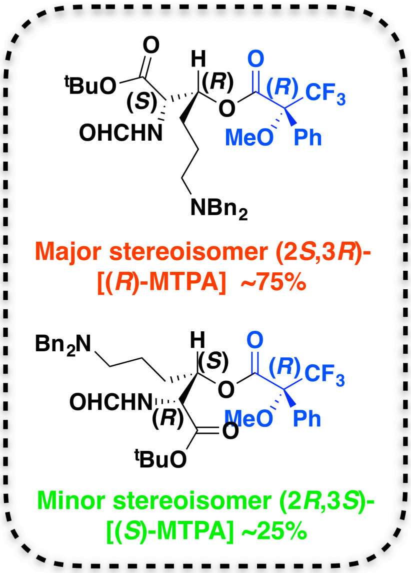

Resolved proton signals	Major stereoisomer of (9)-[(*S*)-MTPA] ^{1}H NMR δ^{S} (ppm)	Major stereoisomer of (9)-[(*R*)-MTPA] ^{1}H NMR δ^{R} (ppm)	$\Delta\delta^{SR} = (\delta^{S} - \delta^{R})$ / (ppm)
-C*H*O	8.15	8.17	-0.02
-C^{2}N*H*CHO	5.91	6.03	-0.12
-C^{3}*H*O-MTPA	5.48	5.52	-0.04
-$C^{6}H_{2}NBn_{2}$	2.37	2.30	+0.07

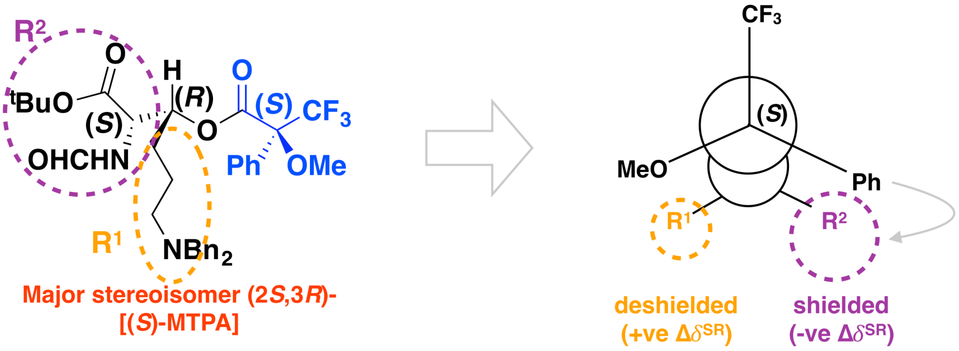

The phenyl group of (*S*)-MTPA shields protons residing in the R^2 **region**. Therefore these protons resonate more upfield compared to the (*R*)-MTPA ester.

Figure S1C ^{1}H NMR Mosher ester analysis of the C-3 alcohol of formamide (9). [3] $\Delta\delta^{SR}$ values obtained from analysis of ^{1}H NMR (400 MHz) spectra of (*S*)- and (*R*)-Mosher's ester derivatives, (**9**)-[(*S*)-MTPA] and (**9**)-[(*R*)-MTPA]. Calculation of $\Delta\delta^{SR}$ values enables assignment of the stereochemistry at C-3 of (**9**). $\Delta\delta^{SR} = (\delta^{S} - \delta^{R})$ i.e. $\Delta\delta^{SR}$ = δ(*S*-MTPA ester) - δ(*R*-MTPA ester).[3] The basis of the Mosher's method is exemplified above for (2*S*,3*R*)-[(*S*)-MTPA].

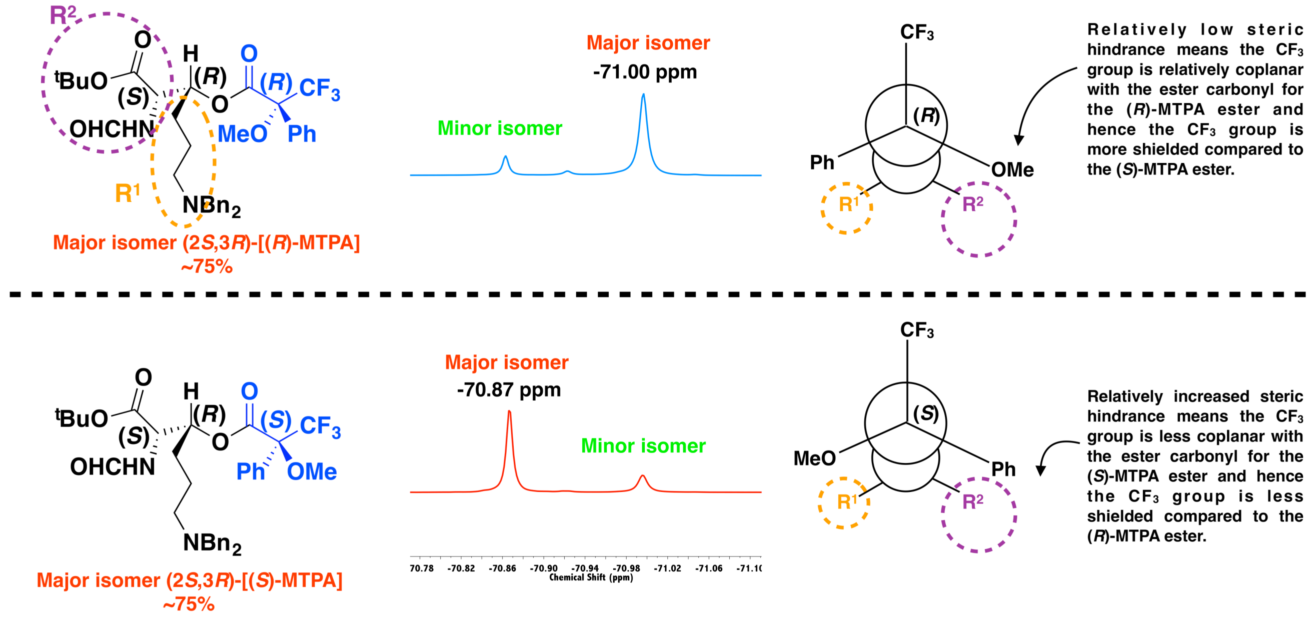

Figure S1D ^{19}F NMR Mosher ester analysis of the C-3 alcohol of formamide (9).[4] Analysis of ^{19}F NMR (377 MHz) spectra of (*S*)- and (*R*)-Mosher's ester derivatives, (**9**)-[(*S*)-MTPA] and (**9**)-[(*R*)-MTPA]. Comparing ^{19}F resonances of both (**9**)-[(*S*)-MTPA] and (**9**)-[(*R*)-MTPA] supports the stereochemical assignment at C-3 of (**9**) as described in Fig. S1C.[4]

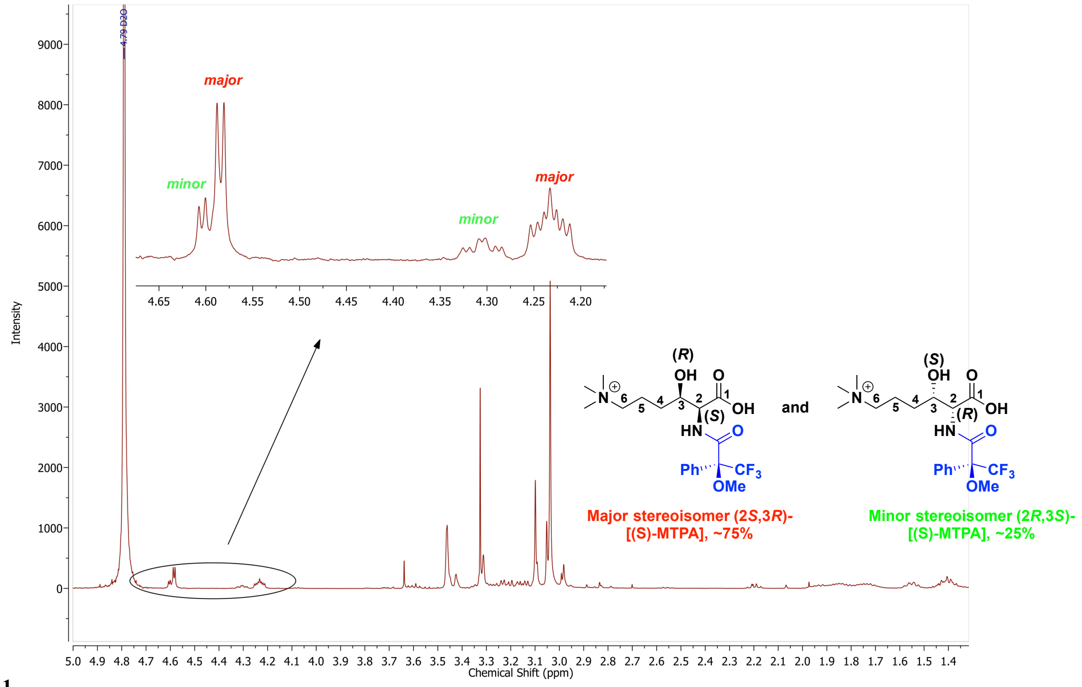

Figure S2 ^{1}H NMR spectrum of the crude aqueous reaction mixture of quaternary amine (13) and (*R*)-Mosher's acid chloride. The signals used in the stereochemical assignment are enlarged.

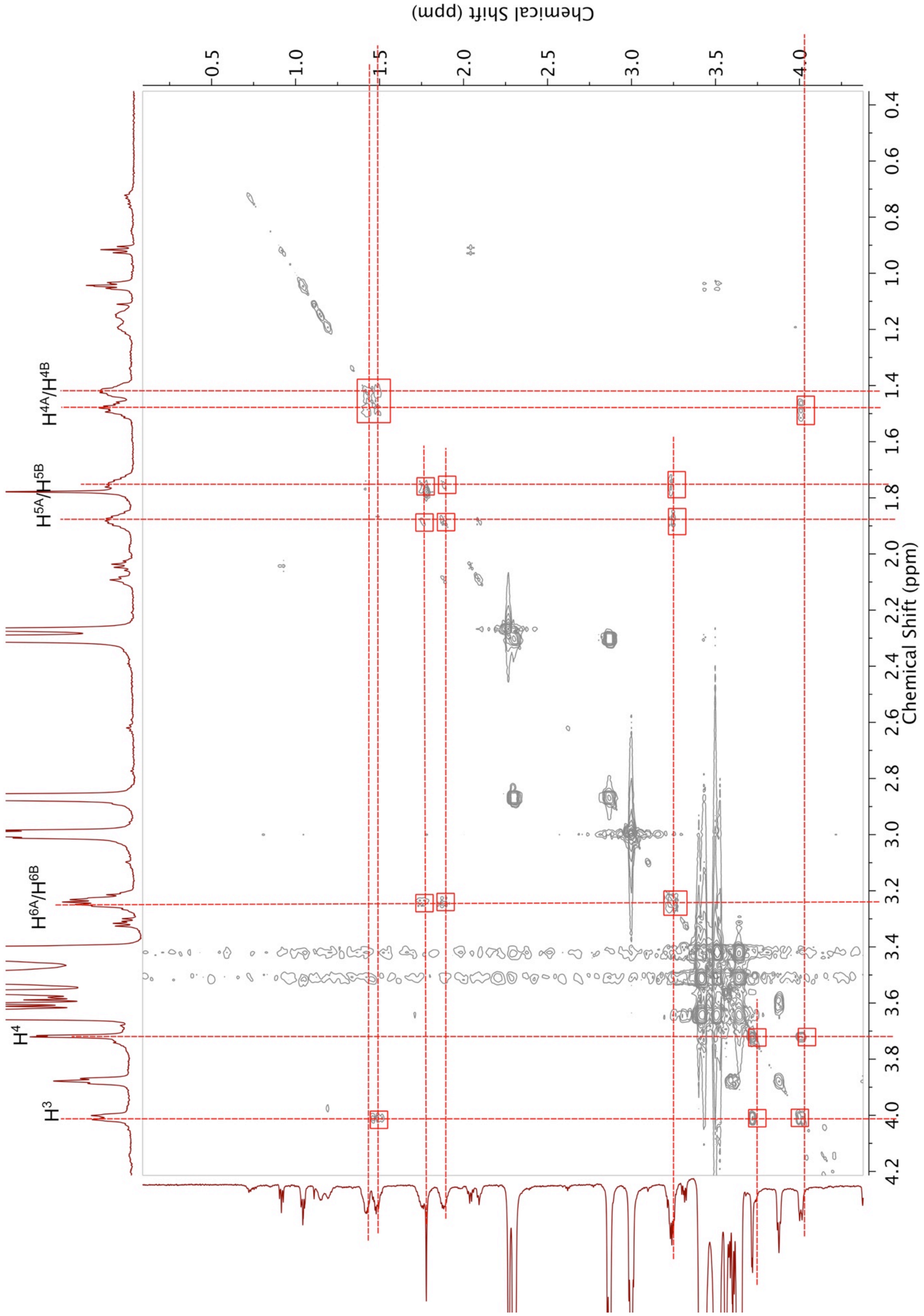

Figure S3A ^1H-^1H COSY spectrum of TMLH-catalysed TML hydroxylation. Reaction conditions: 500 µM TML, 1500 µM 2OG, 500 µM *L*-ascorbate, 150 µM $Fe^{(II)}$, 10 µM TMLH, 50 mM Tris-d_{11}, 200 mM NaCl, pH 7.5.

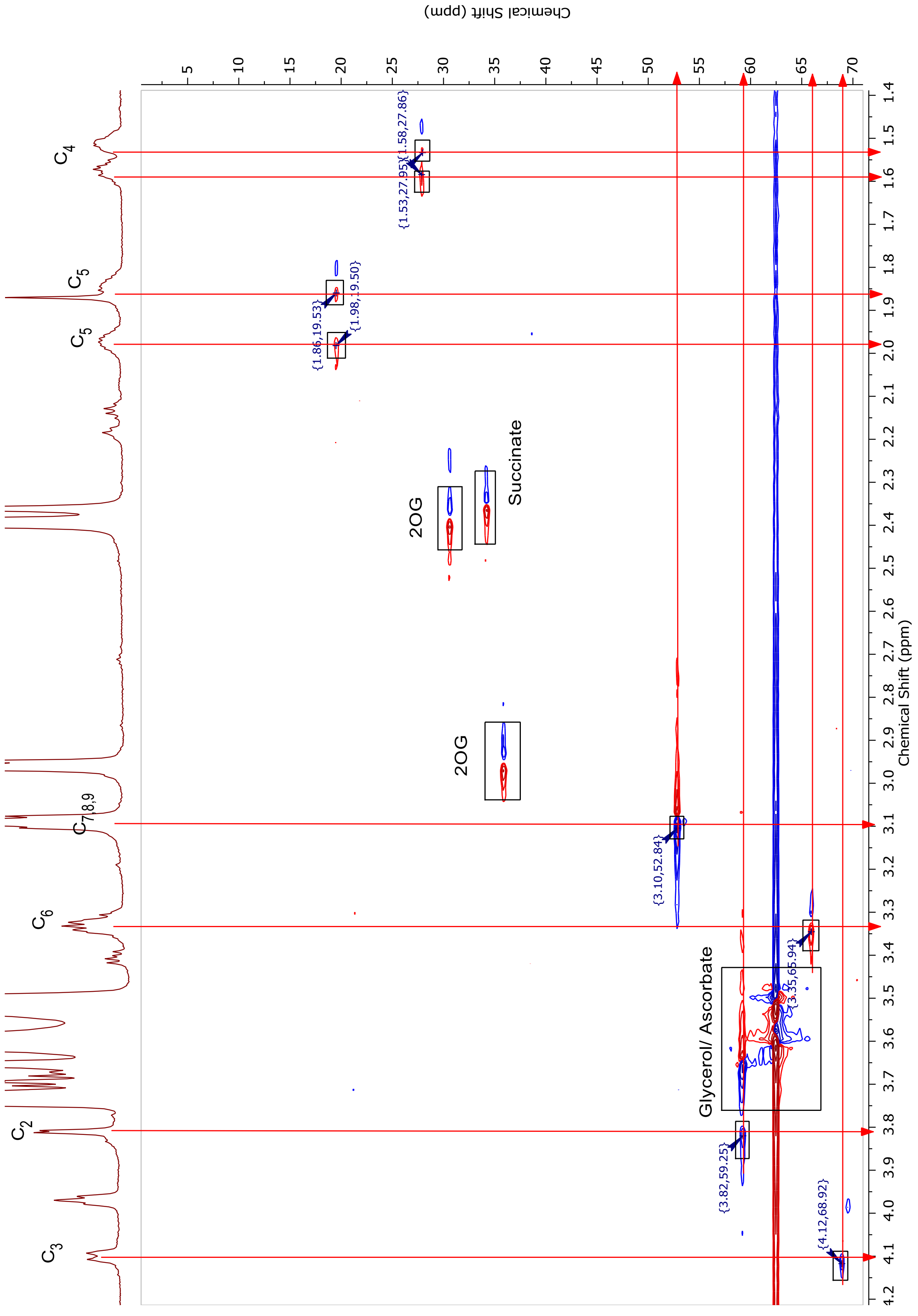

Figure S3B ^{1}H-^{13}C HSQC spectrum of TMLH-catalysed TML hydroxylation. Reaction conditions: 500 µM TML, 1500 µM 2OG, 500 µM *L*-ascorbate, 150 µM $Fe^{(II)}$, 10 µM TMLH, 50 mM Tris-d_{11}, 200 mM NaCl, pH 7.5.

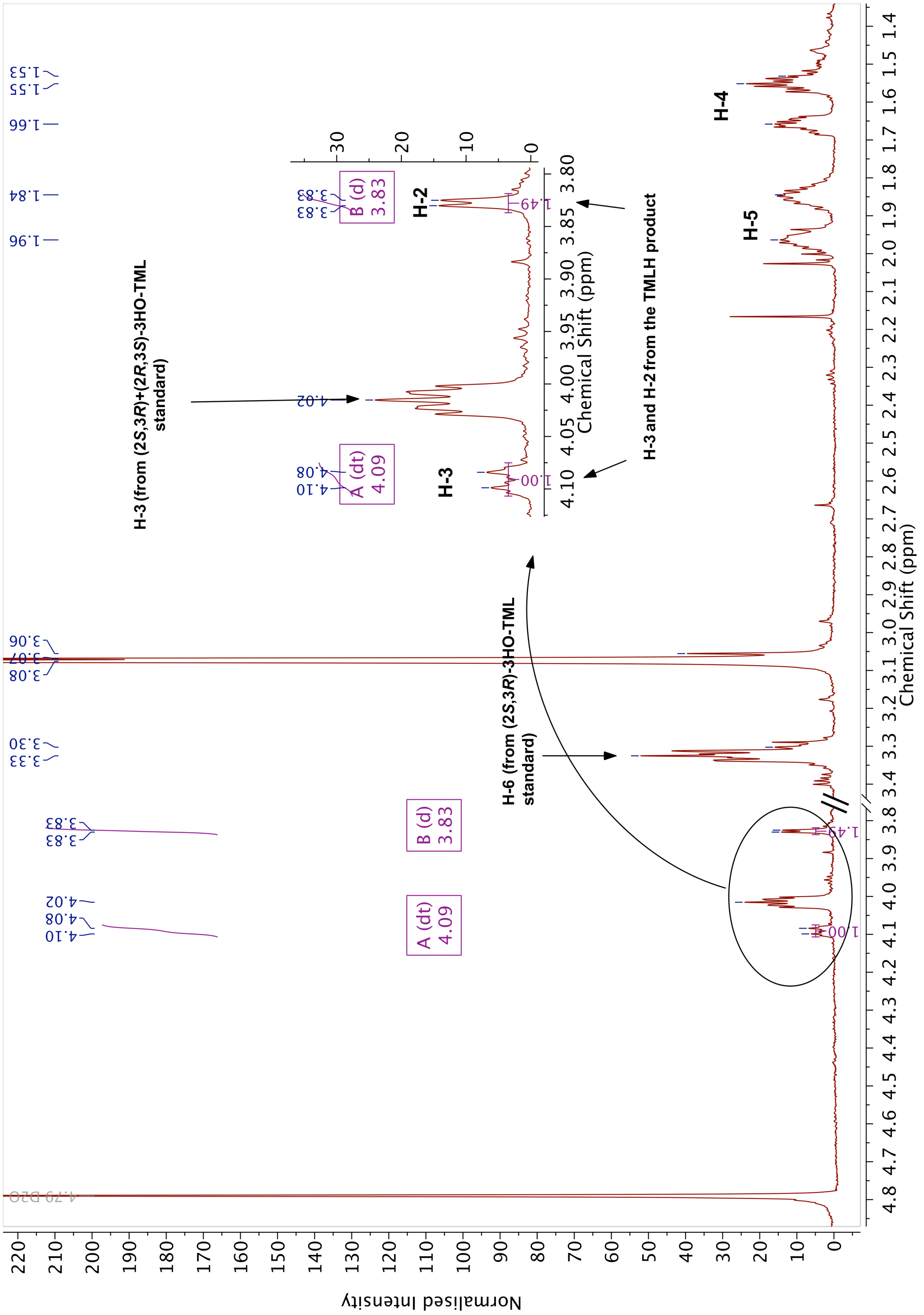

Figure S3C ^{1}H NMR spectrum of TMLH-catalysed (2*S*,3*S*)-3HO-TML (14) spiked with synthetic (2*S*,3*R*)/(2*R*,3*S*)-3HO-TML (13) post-HPLC purification. A TMLH incubation with TML was spiked with synthetic (2*S*,3*R*)-3HO-TML (**13**) and purified by HPLC, as described in the General Experimental Methods section. Signals arising between δ = 3.50 – 3.75 ppm (glycerol and ascorbate) are omitted for clarity.

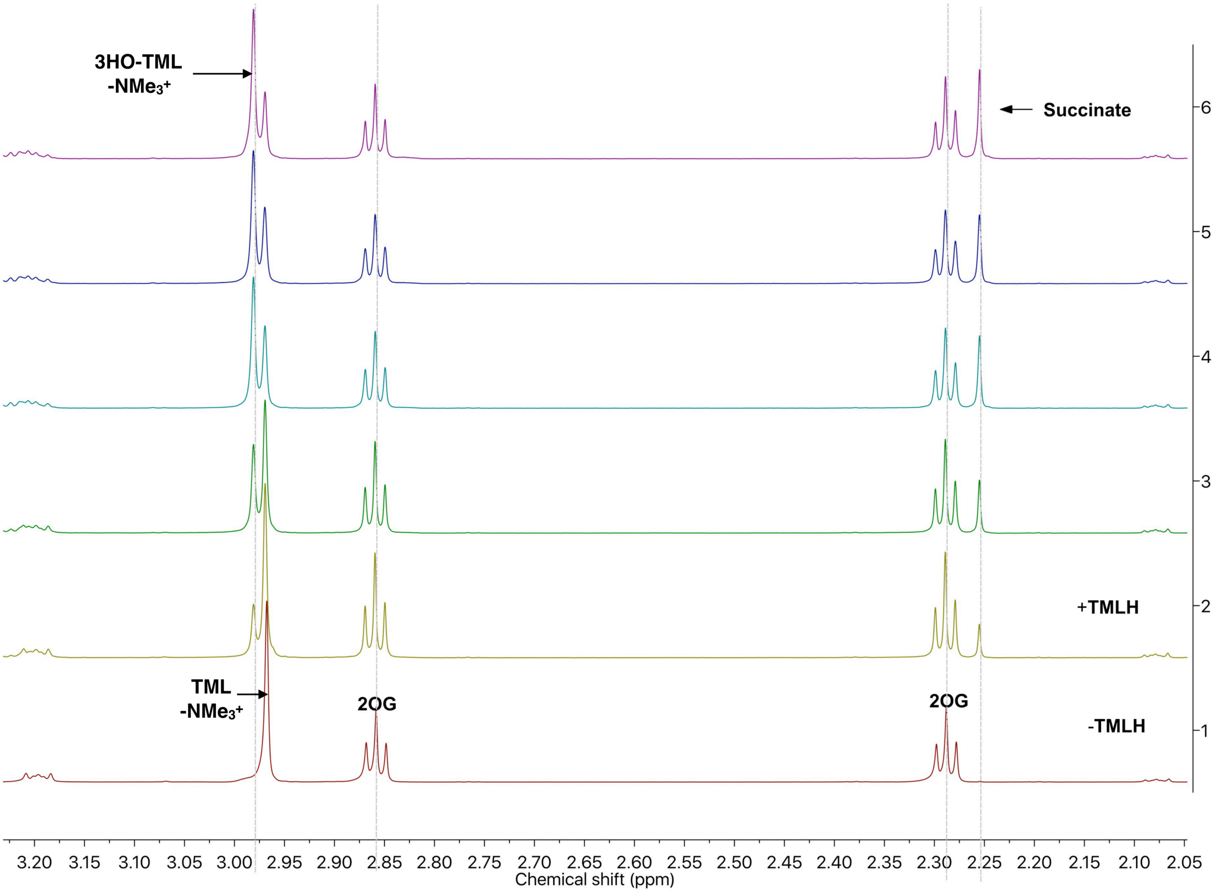

Figure S3D ^{1}H NMR spectra of TMLH-catalysed TML hydroxylation showing coupling of hydroxylation to conversion of 2OG to succinate, monitored over time (δ = 2.05 – 3.20 ppm). Reaction conditions: 500 μM TML, 1500 μM 2OG, 500 μM *L*-ascorbate, 150 μM $Fe^{(II)}$, 10 μM TMLH, 50 mM Tris-d_{11}, 200 mM NaCl, pH 8.0.

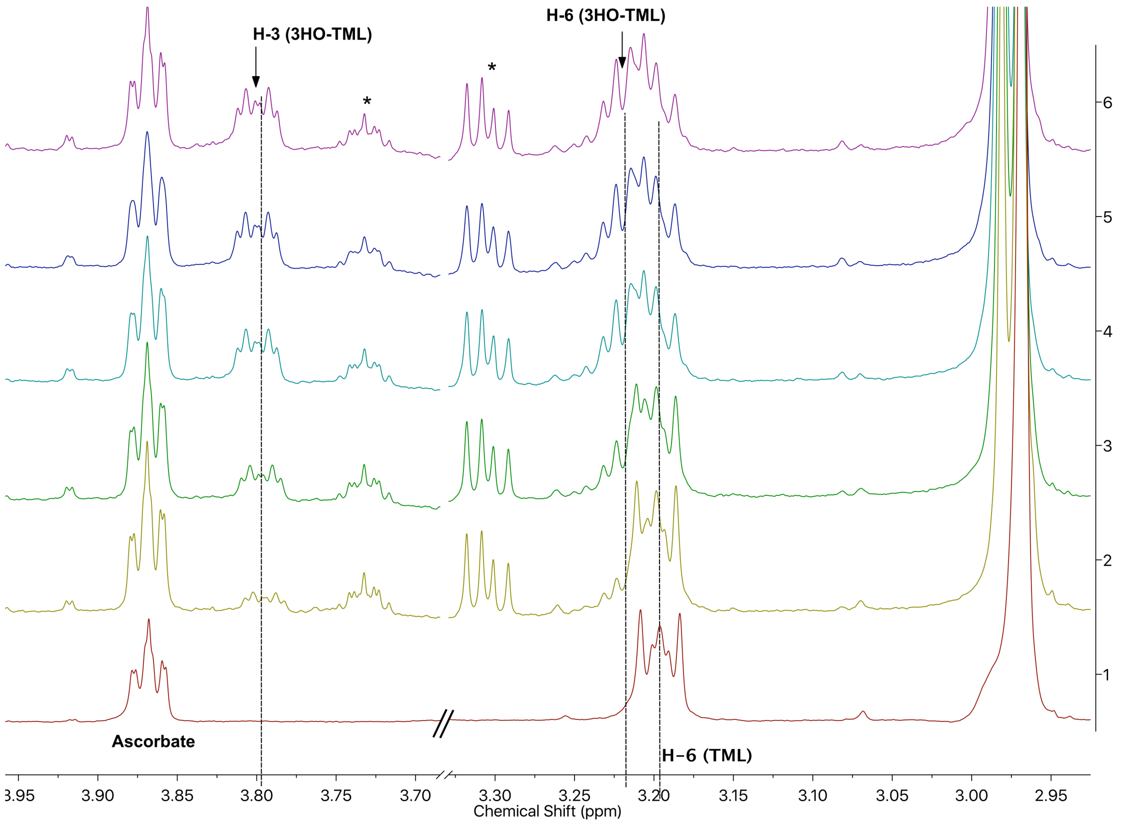

Figure S3E ^{1}H NMR spectra of TMLH-catalysed TML hydroxylation monitored over time (δ = 2.95 – 3.95 ppm). Reaction conditions: 500 µM TML, 1500 µM 2OG, 500 µM *L*-ascorbate, 150 µM $Fe^{(II)}$, 10 µM TMLH, 50 mM Tris-d_{11}, 200 mM NaCl, pH 8.0. Signals between δ = 3.5 – 3.65 ppm (mainly glycerol) have been omitted for clarity. Signals marked with an (*) are from the protein solution.

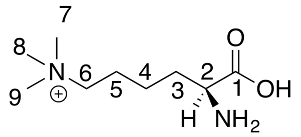

(2*S*)-*N*$^{\varepsilon}$-trimethyllysine (**1**)

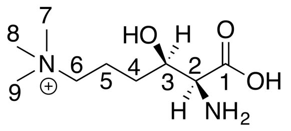

(2*S*,3*R*)-3-hydroxy-*N*$^{\varepsilon}$-trimethyllysine
(Synthesised 3HO-TML) (**13**)

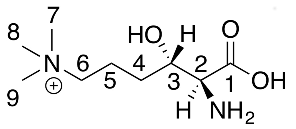

(2*S*,3*S*)-3-hydroxy-*N*$^{\varepsilon}$-trimethyllysine
(TMLH product) (**14**)

Figure S3F Numbering used in TMLH product assignment.

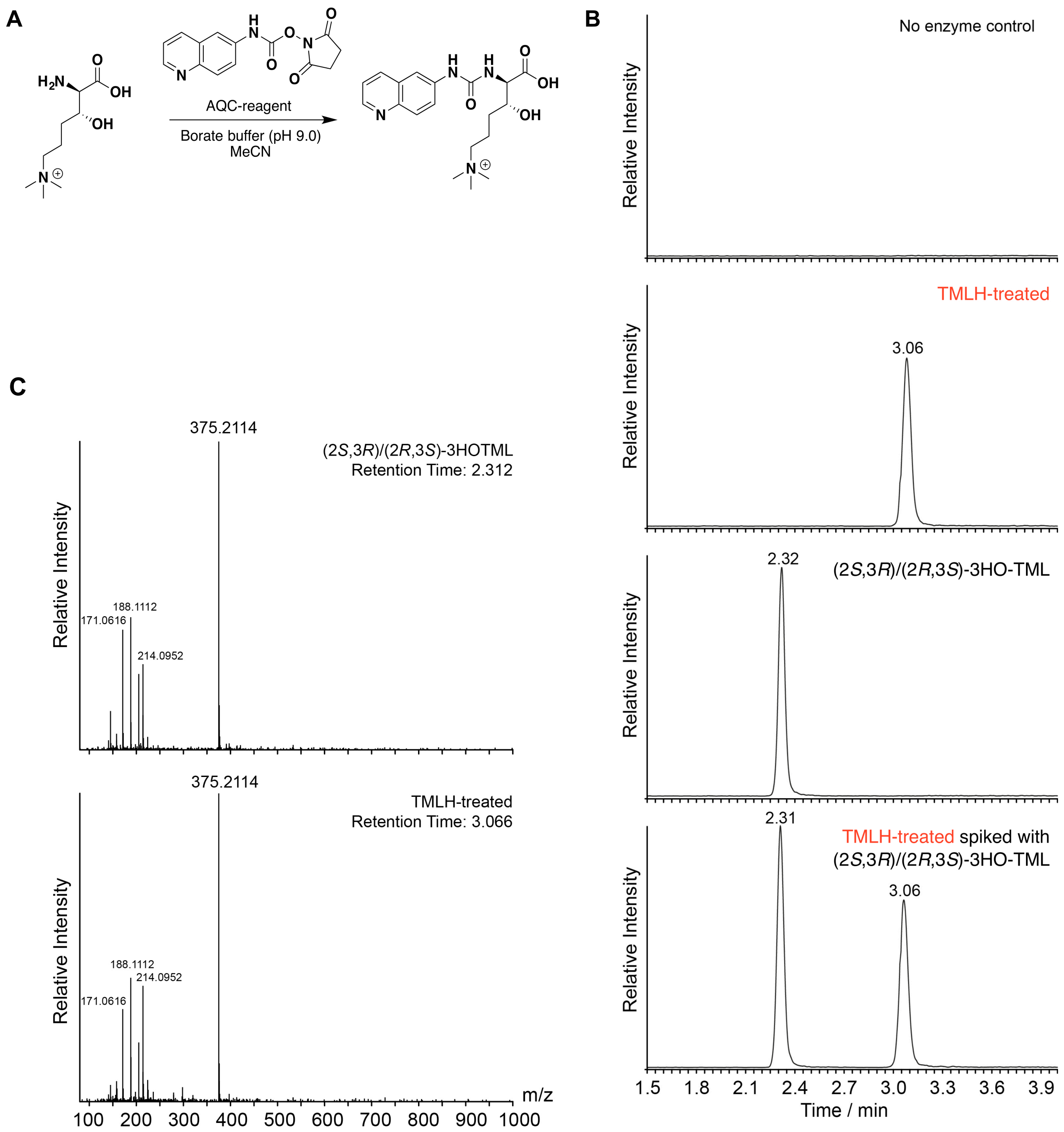

Figure S4 Amino acid analysis of the TMLH-catalysed hydroxylated product. *(A)* Schematic representation of derivatisation of the standard/product for amino acid analysis. *(B)* Non-overlaid extracted ion chromatograms (m/z = 375.2) corresponding to the theoretical exact mass of AQC-derivatised 3HO-TML. *(C)* Resultant mass spectra from (*B*) TMLH-treated sample spiked with (2*S*,3*R*)/(2*R*,3*S*)-3HO-TML at the retention times corresponding to (*top*) the (2*S*,3*R*)/(2*R*,3*S*)-3HO-TML standard (time = 3.214 min) and (*bottom*) the TMLH-treated sample (time = 3.066 min). Note the masses of the derivatised synthetic standard and the TMLH-catalysed product are the same (observed m/z = 375.2114; theoretical m/z = 375.2032).

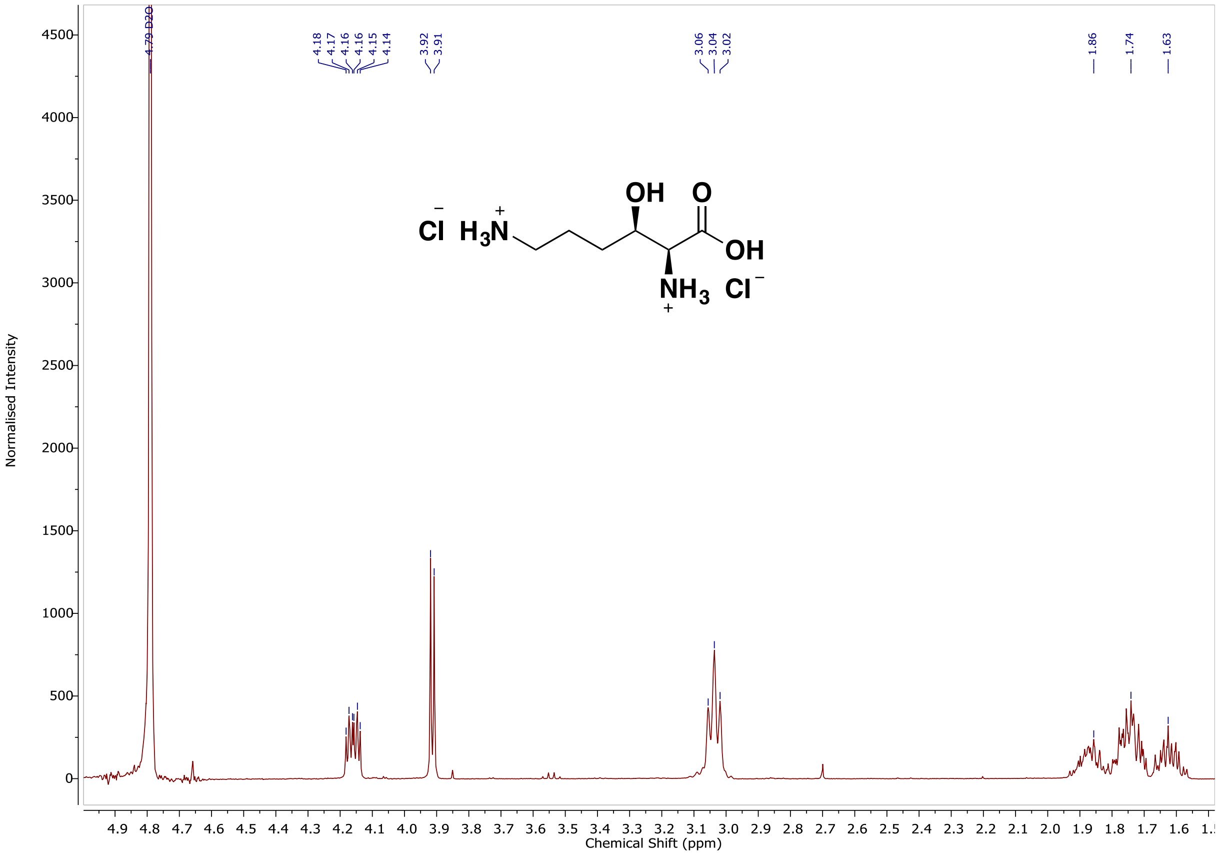

Figure S5A ^{1}H NMR spectrum of (2*S*,3*R*)/(2*R*,3*S*)-3HO-Lys (11).

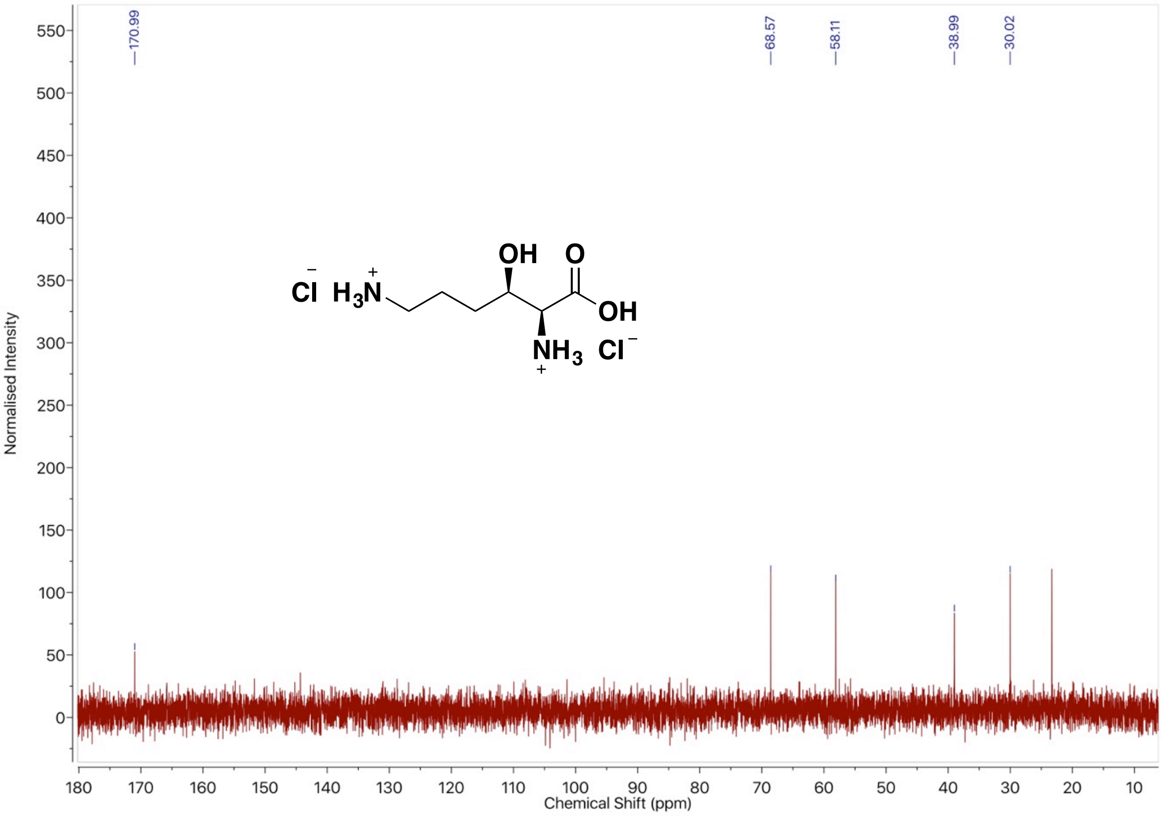

Figure S5B ^{13}C NMR spectrum of (2*S*,3*R*)/(2*R*,3*S*)-3HO-Lys (11).

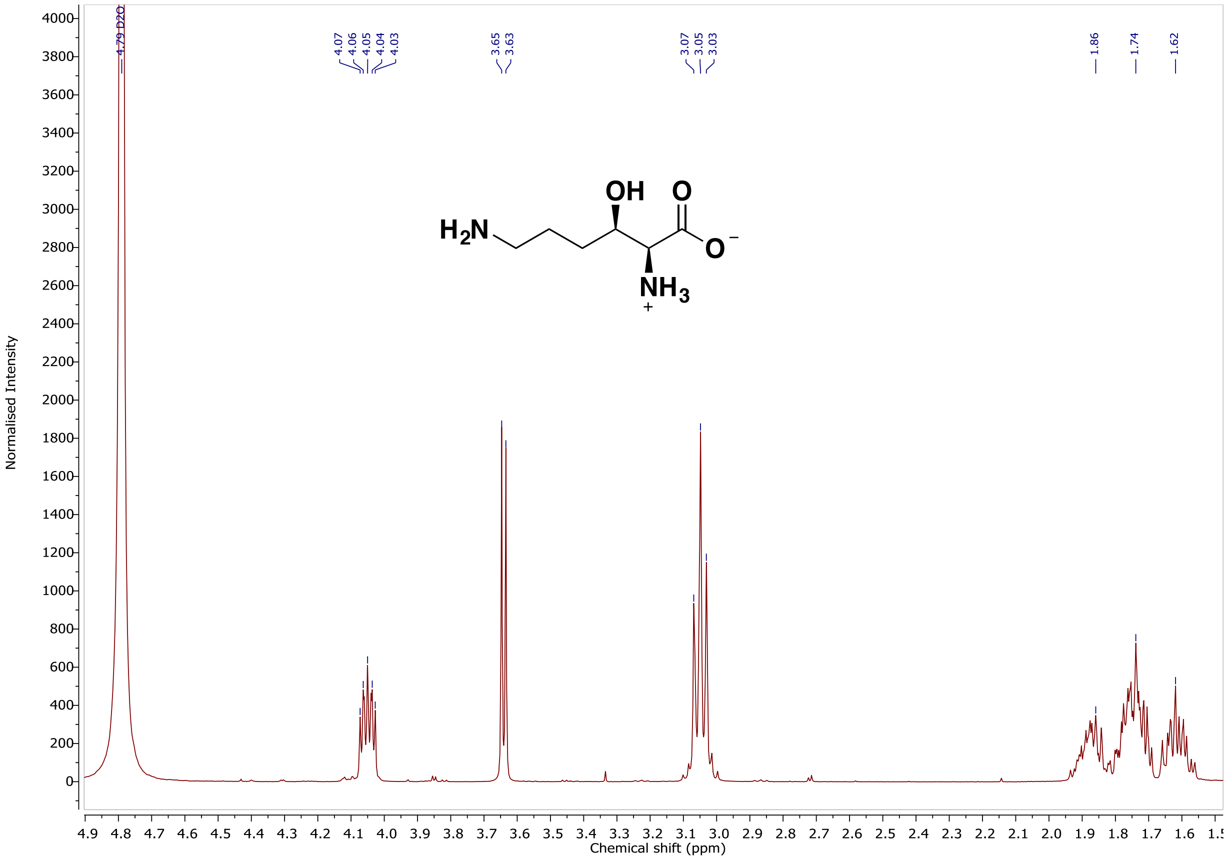

Figure S6A [1]**H NMR spectrum of (2*S*,3*R*)/(2*R*,3*S*)-3HO-Lys (11).**

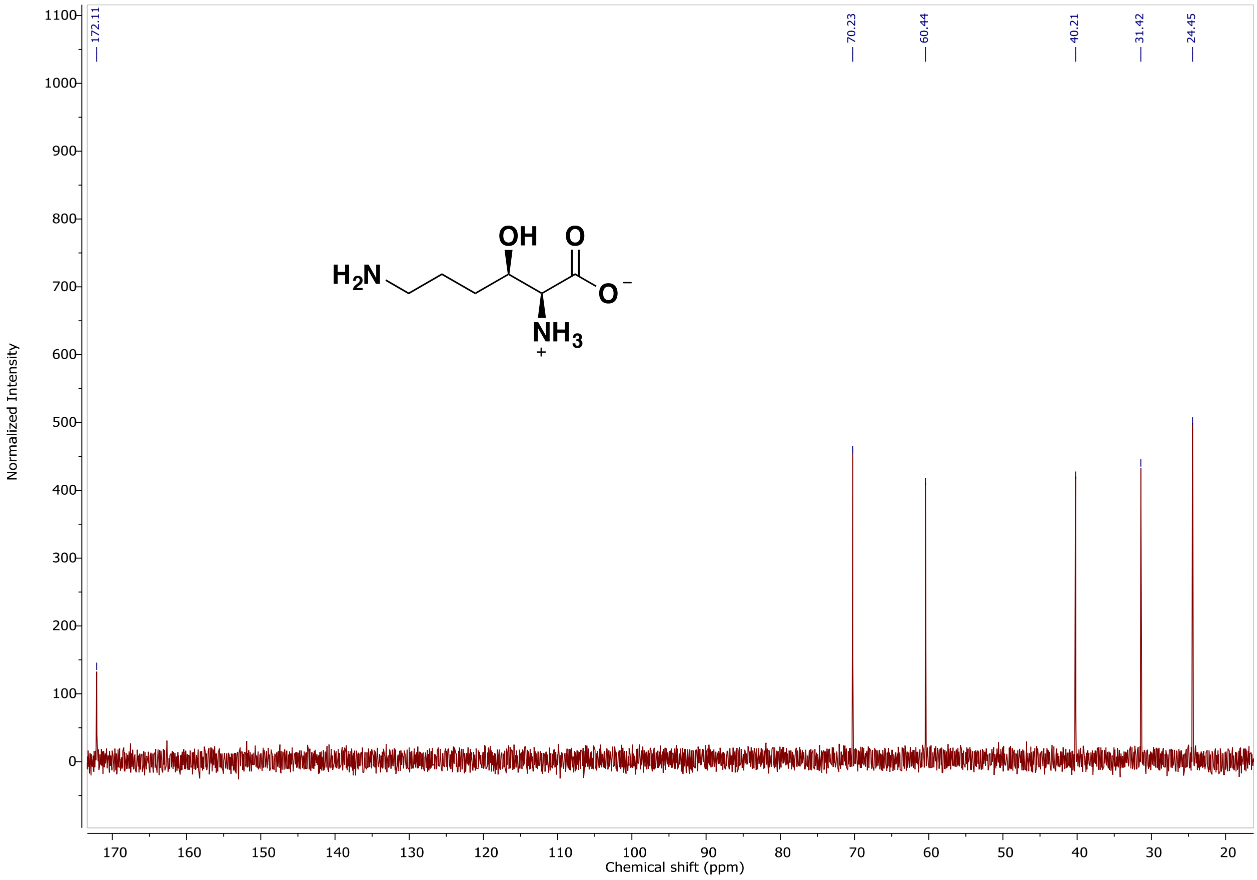

Figure S6B [13]**C NMR spectrum of (2*S*,3*R*)/(2*R*,3*S*)-3HO-Lys (11).**

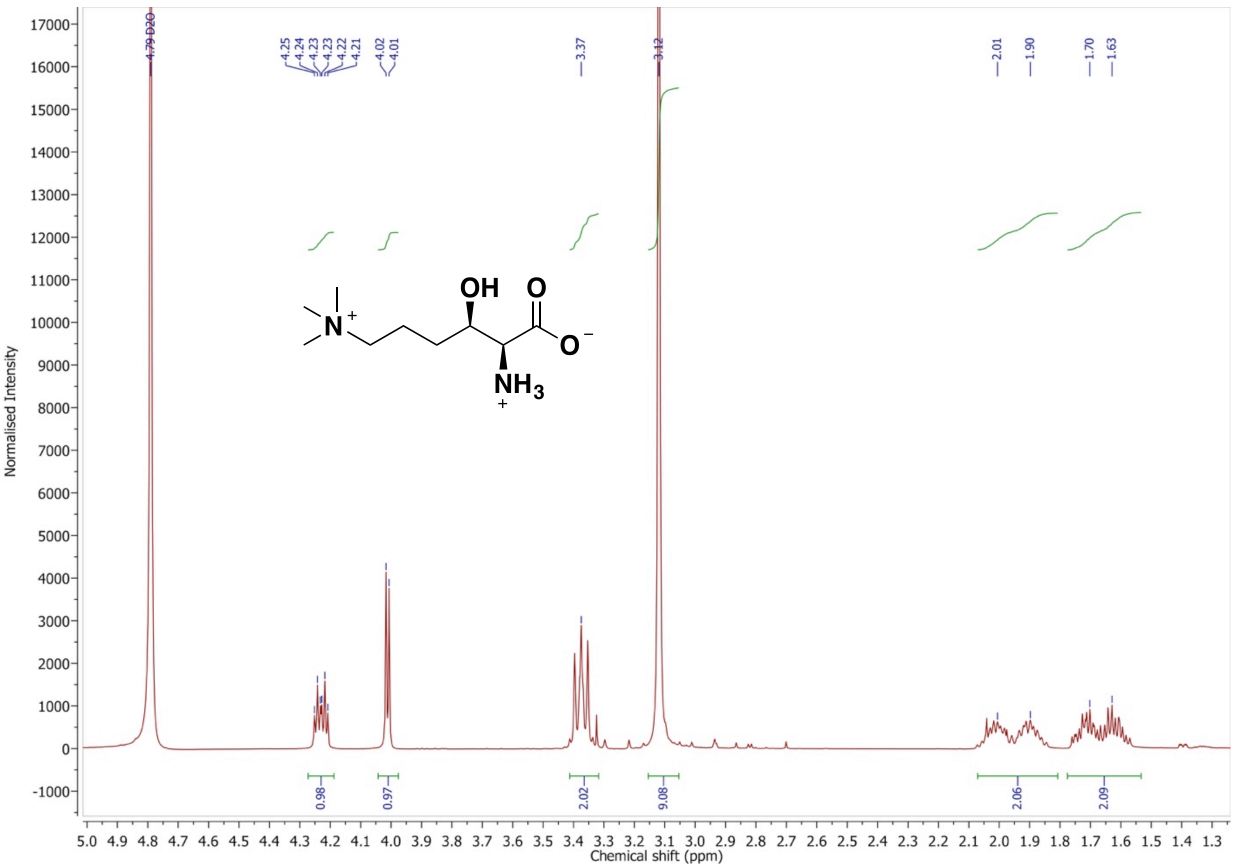

Figure S7A ^{1}**H NMR spectrum of (2*S*,3*R*)/(2*R*,3*S*)-3HO-TML (13).**

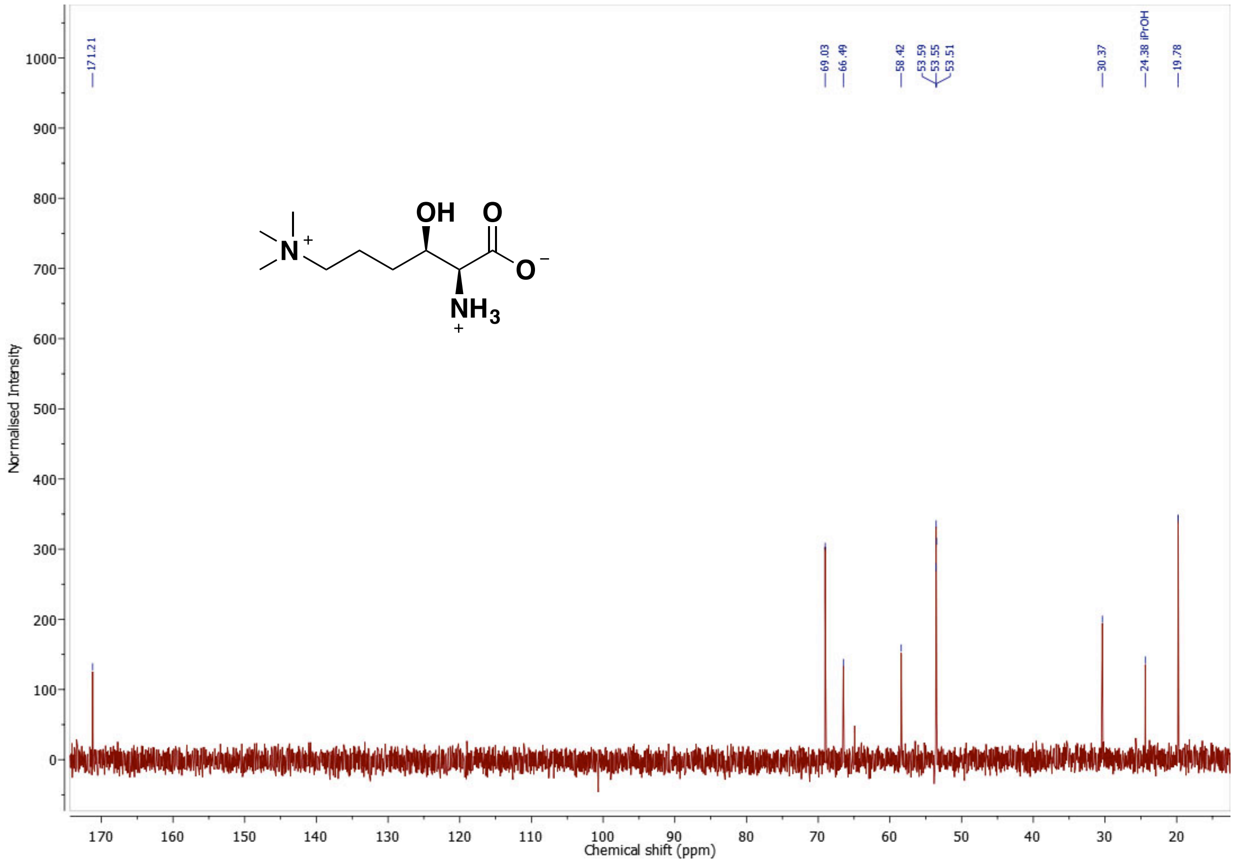

Figure S7B ^{13}**C NMR spectrum of (2*S*,3*R*)/(2*R*,3*S*)-3HO-TML (13).**

Synthesis

General Considerations

All chemicals, reagents, and solvents were obtained from Sigma-Aldrich (Dorset, UK) and used without further purification. HPLC grade solvents were used for reactions, chromatography, and work-ups. Aqueous solutions were made using de-ionized water. Thin layer chromatography (TLC) was carried out using Merck (Darmstadt, Germany) silica gel 60 F254 TLC plates. TLC visualisation was carried out under UV light and stained with one of three stains; ninhydrin, potassium permanganate, or anisaldehyde. Chromatographic purifications were carried out using a Biotage® (Uppsala, Sweden) Isolera One or Biotage® SP4 flash purification system, using Biotage® pre-packed SNAP columns. Reactions were monitored using an Agilent (Cheshire, UK) 1200 series, 6120 quadrupole LC-MS system, and a Merck Chromolith® Performance RP-18 HPLC column. Deuterated solvents were obtained from Sigma-Aldrich, and 1D and 2D NMR spectra were obtained on a Bruker AVIII HD 400 (400 MHz), Bruker AVII 500 (500 MHz) with a ^{13}C cryoprobe, and/or Bruker AVIII 700 (700 MHz) with an inverse TCI cryoprobe. All signals are described in δ ppm with multiplets being denoted as singlet, doublet, triplet, quartet, and multiplet using the abbreviations s, d, t, q, and m, respectively. Chemical shifts were referenced using residual solvent peaks with coupling constants, *J*, reported in hertz (Hz) to an accuracy of 0.5 Hz. For high-resolution mass spectrometry (HR-MS), a Bruker MicroTOF instrument with an ESI source and Time of Flight (TOF) analyser was used. MS data are represented as a ratio of mass to charge (m/z) in Daltons. A Bruker Tensor 27 instrument was used to obtain Fourier transform infrared spectra (FT-IR). Spectroscopic grade solvents and a Perkin Elmer 241 Polarimeter were used to obtain optical rotations.

Dibenzyl-4,4-diethoxybutan-1-amine

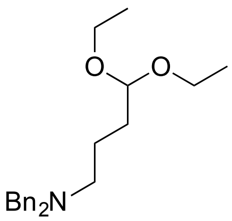

4-Aminobutyraldehyde diethyl acetal (**5**) (12.4 ml, 71.5 mmol) was dissolved in a 4:1 (v/v) of EtOH:distilled water (70 ml). Sodium carbonate (22.73 g, 214.5 mmol) was then added, followed by a solution of benzyl bromide (29.8 ml, 250.3 mmol) in EtOH (14 ml), which was added dropwise over 30 minutes. The resultant mixture was left under reflux overnight. The solvent was then removed *in vacuo,* and the resulting material diluted with water (60 ml) and CH_2Cl_2 (30 ml). The aqueous layer was washed with CH_2Cl_2 (2 x 30 ml); the organic layers were combined, dried over $MgSO_4$, then filtered. After evaporation of the solvent *in vacuo,* the crude reaction mixture was loaded onto a silica column and purified using flash column chromatography with an isocratic gradient (50:50 EtOAc/cyclohexane). After collecting the appropriate fractions, the solvent was removed *in vacuo* to yield dibenzyl-4,4-diethoxybutan-1-amine as a thin, colourless oil (21.8 g, 63.9 mmol, 89%).

^{1}H NMR (400 MHz, chloroform-*d*) δ = 7.4 – 7.2 (10H, m, *Ph*), 4.4 – 4.3 (1H, m, C*H*O_2Et_2), 3.7 – 3.3 (m, 8H, OC*H*$_2$CH$_3$, C*H*$_2$Ph), 2.4 (2H, t, *J*=6.5 Hz, NC*H*$_2$CH$_2$), 1.7 – 1.5 (4H, m, NCH$_2$C*H*$_2$C*H*$_2$), 1.2 (6H, t, *J*=7.0 Hz, OCH$_2$C*H*$_3$) ppm. **^{13}C NMR** (101 MHz, chloroform-*d*) δ = 140.0, 128.9, 128.3, 126.9, 102.9, 61.1, 53.1, 31.2, 22.3, 15.5 ppm. **FT-IR** ν_{max} (film) 3028, 2973, 2928, 2874, 2795, 1494, 1453, 1372, 1124, 1060, 743, 698 cm^{-1}. **HRMS** (ESI-TOF) calcd for $C_{22}H_{32}NO_2$ [M+H]$^+$: 342.2428, found: 342.2429.

4-(dibenzylamino)butanal (6)

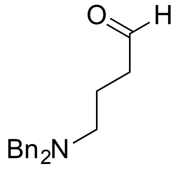

Dibenzyl-4,4-diethoxybutan-1-amine (2.0 g, 5.87 mmol) was dissolved in THF (2.93 ml); 20% (v/v) $HCl_{(aq)}$ solution (2.93 ml) was then added. This mixture was stirred for 1 h at room temperature until complete reaction was observed by TLC (4:1 cyclohexane/EtOAc). The reaction was quenched by the addition of saturated $NaHCO_{3(aq)}$. The organic material was then extracted with EtOAc (3 x 20ml); the combined organic layers were washed with saturated $NaHCO_3$ and brine, then dried over $MgSO_4$ and filtered. After removal of volatiles by evaporation *in vacuo*, the crude reaction material was purified via flash column chromatography (0-20% cyclohexane/EtOAc over ~10 column volumes). The appropriate fractions were combined and the solvent removed to yield (**6**) as a pale yellow oil (1.13 g, 4.23 mmol, 72%).

^{1}**H NMR** (400 MHz, chloroform-*d*) δ = 9.7 (1H, t, *J*=1.5 Hz, C*H*O), 7.3 (10H, s, *Ph*), 3.6 (4H, s, NCH_2Ph), 2.5 – 2.4 (4H, m, NCH_2CH$_2$CH_2), 1.8 (2H, p, *J*=7.0 Hz, NCH$_2$CH_2CH$_2$) ppm. ^{13}**C NMR** (101 MHz, chloroform-*d*) δ = 202.7, 139.6, 128.9, 128.3, 127.0, 58.5, 52.5, 41.8, 20.0 ppm. **FT-IR** ν_{max} (film) 3027, 2802, 1721, 1493, 1452, 745, 698 cm^{-1}. **HRMS** (ESI-TOF) calcd for $C_{18}H_{22}NO$ $[M+H]^+$: 268.1696, found: 268.1693.

N-[(9*R*)-10,11-dihydrocinchonan-9-yl]-2-(diphenylphosphino)benzamide (7)[5]

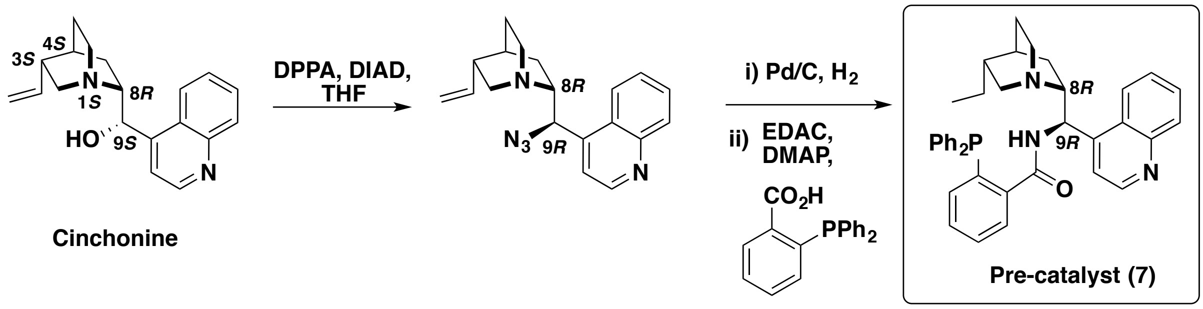

The procedures of Sladojevich *et al.*[5] and Cassani *et al.*[6] were used. Cinchonine[7] (3.0 g, 10.2 mmol) and triphenylphosphine (3.2 g, 12.2 mmol) were suspended in dry THF (51 ml). The mixture was cooled in an ice bath to 0°C, after which diisopropyl azodicarboxylate (2.41 ml, 12.2 mmol) was added. Diphenyl phosphoryl azide (2.64 ml, 12.2 mmol) was then dissolved in dry THF (21 ml); the resultant solution was added dropwise to the reaction mixture. Upon removing

the ice bath, the reaction mixture was allowed to warm to room temperature and stirred overnight. The resultant solution was heated for 2 h at 50°C, after which the volatiles were removed *in vacuo*. The resultant crude mixture was dissolved in CH_2Cl_2 (150 ml), and 4M hydrochloric acid was added. The aqueous phase was removed, and solid $NaHCO_3$ was then added to the aqueous phase until the pH was ~10 (litmus paper). The basic aqueous phase was then extracted with CH_2Cl_2 (3 x 100 ml), dried over $MgSO_4$, the CH_2Cl_2 was then removed *in vacuo* to give the desired azide in 94% crude yield.

The crude azide was then dissolved in MeOH (50 ml), and 10% Pd/C (750 mg) was added. The reaction flask was placed under a H_2 atmosphere by fitting the vessel with a septum, H_2 balloon, and syringe. The flask was evacuated and filled with H_2 three times, then stirred at room temperature at 1 atm overnight. The reaction mixture was then filtered through a short pad of Celite®; volatiles were removed *in vacuo* to yield the crude amine in apparent quantitative yield as a yellow oil, which was used without further purification.

The crude amine (1.70 g, 5.7 mmol) was dissolved in anhydrous CH_2Cl_2 (50 ml). 2-(diphenylphosphino)-benzoic acid (1.94 g, 6.3 mmol) and 4-dimethylaminopyridine (7 mg, 0.057 mmol) were then added. The resultant mixture was cooled to 0°C, then *N*-(3-dimethylaminopropyl)-*N'*-ethylcarbodiimide hydrochloride (1.1 g, 5.7 mmol) was added. The reaction mixture was allowed to warm to room temperature, after which it was stirred overnight at room temperature. After removing volatiles *in vacuo*, the resultant crude mixture was dissolved in EtOAc (50 ml), then washed with water, 10% $NaHCO_{3(aq)}$, and brine. After drying the organic phase over Na_2SO_4 and removing the solvent *in vacuo*, the crude mixture was purified by flash chromatography using an isocratic elution system (EtOAc/CH_2Cl_2/MeOH 5:2:1) to yield the desired compound as a pale yellow foam (3.2 g, 5.51 mmol, 54%, over three steps).

^{1}H NMR (400 MHz, chloroform-*d*) δ = 8.75 (1H, d, *J*=4.5 Hz), 8.38 (1H, d, *J*=8.5 Hz), 8.11 (1H, dd, *J*=8.5, 1.5 Hz), 7.74 – 7.55 (4H, m), 7.40 – 7.15 (13H, m), 6.89 (1H, dd, *J*=7.0, 4.0 Hz), 5.37 (1H, s), 3.05 – 2.63 (4H, m), 2.59 – 2.41 (1H, m), 1.52 (1H, s), 1.47 – 1.32 (5H, m), 1.25 – 1.18 (1H, m), 0.89 (3H, t, *J*=7.0 Hz), 0.89 – 0.80 (1H, m) ppm. **^{13}C NMR** (101 MHz, chloroform-*d*) δ = 169.1, 150.2, 148.5, 146.7, 141.3, 141.0, 137.8, 137.7, 137.3, 137.1, 135.9, 135.7, 134.3, 133.9, 133.8, 133.7, 133.6, 130.3, 127.4, 126.6, 123.5, 119.6, 60.5, 49.0, 37.3, 27.1, 26.0, 25.7, 25.0, 12.1 ppm. **^{31}P NMR** (162 MHz, chloroform-*d*) δ = -10.3 ppm. **FT-IR** ν_{max} (film) 2980, 1654, 1457, 745, 697 cm^{-1}. $[\alpha]_D^{25}$ = + 60.6 (c = 0.2 in CH_2Cl_2)(Lit $[\alpha]_D^{25}$ = +67.2, c = 0.5 CH_2Cl_2). **HRMS** (ESI-TOF): calcd for $C_{38}H_{39}N_3O_1P_1$ $[M+H]^+$ 584.2825, found 584.2825. Spectroscopic data are consistent with those reported.[5]

tert-Butyl (2*S*,3*R*)-3-(6-(dibenzylamino)propyl)-2,3-dihydrooxazole-2-carboxylate (8)

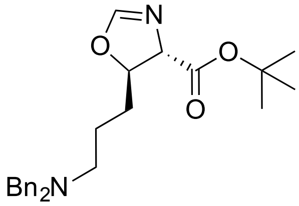

Pre-catalyst (**7**)[3] (53 mg, 0.09 mmol) was dissolved in anhydrous EtOAc (5 ml) prior to addition of Ag_2O (9.3 mg, 0.04 mmol). The mixture was stirred for ~5 min at room temperature, then cooled in a dry ice/acetone bath. On cooling, *tert*-butyl isocyanoacetate (0.22 ml, 1.47 mmol) was added, followed by crushed, dried 4Å molecular sieves. A solution of aldehyde (**6**) (470 mg, 1.76 mmol) in anhydrous EtOAc (1 ml) was added; the reaction was quickly placed in a -20°C freezer with occasional stirring. The reaction was left overnight; consumption of the isocyanoacetate was monitored by TLC (50:50 EtOAc/cyclohexane), staining with anisaldehyde solution. After 16 h, the reaction mixture was quickly filtered through a short pad of silica gel eluting with EtOAc. The filtered reaction mixture was concentrated *in vacuo*, then purified using silica flash chromatography (0–40% EtOAc/cyclohexane over ~10 column volumes). Concentration of the appropriate fractions gave oxazoline (**8**) (468 mg, 1.15 mmol, 78%, d.r. > 95:1) as a viscous, colourless oil. An e.r. of 3:1 was determined by analysis of the ^{1}H NMR spectra of the corresponding Mosher's ester of formamide (**9**) (Fig. S1).

^{1}H NMR (500 MHz, toluene-d_8) δ = 7.32 – 7.26 (m, 4H, *Ph*), 7.19 – 7.14 (m, 4H, *Ph*), 7.11 – 7.07 (m, 2H, *Ph*), 6.36 (d, *J*=2.0, 1H, OC*H*N), 4.62 – 4.54 (m, 1H, $CH_2C\mathit{H}$(O)CH(N)), 4.06 (dd, *J* =7.0, 2.1, 1H, CH_2CH(O)C*H*(N)), 3.40 – 3.31 (m, 4H, $NC\mathit{H_2}Ph$), 2.24 (t, *J*=6.5, 2H, $NC\mathit{H_2}CH_2$), 1.49 – 1.36 (m, 3H, $NCH_2C\mathit{H_2}C\mathit{H_A}$), 1.35 (s, 9H, *^{t}Bu*), 1.29 – 1.20 (m, 1H, $NCH_2CH_2C\mathit{H_B}$) ppm. **^{13}C NMR** (126 MHz, toluene-d_8) δ = 170.11, 155.58, 140.08, 129.04, 128.49, 127.18, 81.50, 81.06, 74.12, 58.73, 53.12, 32.78, 27.86, 22.78 ppm. **FT-IR** ν_{max} (film) 3027, 2979, 2797, 1732, 1624, 1367, 1155, 1110, 745, 698 cm^{-1}. $[\alpha]_D^{25}$ = + 66.2° (c = 2.65 in CH_2Cl_2). **HRMS** (ESI-TOF): calcd for $C_{25}H_{32}O_3N_2Na_1$ $[M+Na]^+$ 431.2305, found 431.2306.

tert-Butyl (2*S*,3*R*)-6-(dibenzylamino)-2-formamido-3-hydroxyhexanoate (9)

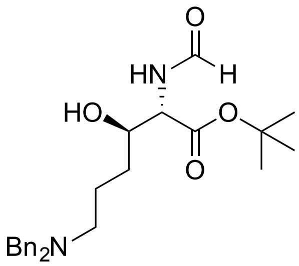

tert-Butyl(2*S*,3*R*)-3-(6-(dibenzylamino)propyl)-2,3-dihydrooxazole-2-carboxylate (**8**) (400 mg, 0.98 mmol) was dissolved in THF (6 ml) prior to addition of 20% (v/v) $AcOH_{(aq)}$ (4 ml). This mixture was stirred for 30 min (TLC monitor, 100% EtOAc) before the addition of saturated aqueous sodium hydrogen carbonate until a pH ~ 8 was reached. CH_2Cl_2 was then added and the organic material extracted with CH_2Cl_2 (3 x 20 ml). The organic layers were combined, dried over $MgSO_4$, and then evaporated *in vacuo*. The crude reaction material was purified using flash chromatography (0-100% EtOAc/cyclohexane over ~10 column volumes). The appropriate fractions were combined, and the solvent removed *in vacuo* to yield formamide (**9**) as a clear, viscous oil (379 mg, 0.89 mmol, 91%).

^{1}H NMR (400 MHz, chloroform-*d*) δ = 8.1 (1H, s, NHC*H*O), 7.3 – 7.1 (10H, m, *Ph*), 6.5 (1H, d, *J*=9.0 Hz, CHN*H*CHO), 4.5 (1H, dd, *J*=10.0, 2.0 Hz, C*H*NHCHO), 4.0 (1H, dt, *J*=10.0, 2.0 Hz, C*H*OH), 3.7 (2H, d, *J*=13.0 Hz, NCH_2Ph), 3.3 (2H, d, *J*=13.0 Hz, NCH_2Ph), 2.5 – 2.3 (2H, m, NC$H_2$$CH_2$$CH_2$), 2.0 (1H, s, O*H*), 1.7 – 1.6 (3H, m, NCH_2CH_2CH_A), 1.4 (9H, s, ^{t}*Bu*), 1.2 – 1.1 (1H, m, NCH_2CH_2CH_B) ppm. ^{13}C NMR (101 MHz, chloroform-*d*) δ = 169.8, 161.4, 137.3, 129.8, 128.4, 127.5, 82.2, 72.0, 58.3, 55.8, 54.0, 33.9, 28.1, 24.1 ppm. **FT-IR** ν_{max} (film) 3356, 2934, 1738, 1669, 1369, 1158, 746, 699 cm^{-1}. $[\alpha]_D^{25}$ = + 10.4° (c = 0.414 in CH_2Cl_2). **HRMS** (ESI-TOF): calcd for $C_{25}H_{35}O_4N_2$ $[M+H]^+$ 427.2591, found 427.2577.

tert-Butyl (2*S*,3*R*)-6-amino-2-formamido-3-hydroxyhexanoate (10)

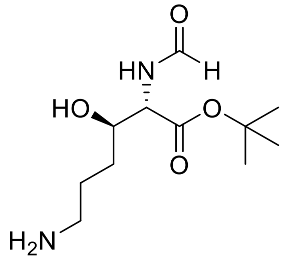

tert-Butyl (2*S*,3*R*)-3-(6-(dibenzylamino)propyl)-2,3-dihydrooxazole-2-carboxylate (**8**) (250 mg, 0.61 mmol) was dissolved in MeOH (5 ml) prior to addition of 5% (v/v) aqueous citric acid (2 ml). 10% palladium on carbon (100 mg/mmol) was added to the solution, and the reaction vessel was subsequently placed under an atmosphere of H_2 and stirred at room temperature for 24 h until complete conversion was observed (by TLC and/or LC-MS). The reaction mixture was filtered through a short pad of Celite®, then evaporated *in vacuo*. The residue was dissolved in CH_2Cl_2 (10 ml) and washed with a solution of saturated $NaHCO_3$ (10 ml). The aqueous fractions were frozen in liquid nitrogen and lyophilized. The resultant material was purified by loading onto a silica column pre-equilibrated with 100% CH_2Cl_2 and eluted with a gradient of 0-70% MeOH over 10 column volumes. The appropriate fractions were then concentrated *in vacuo* to yield amine (**10**) as a colourless oil (145 mg, 0.59 mmol, 96%).

^{1}H NMR (methanol-d_4, 400 MHz) δ = 8.2 (1H, s, NHC*H*O), 4.5 (1H, d, *J*=3.0 Hz, C*H*NHCHO), 4.1 (1H, td, *J*=6.5, 3.0 Hz, C*H*OH), 2.9 (2H, t, *J*=7.5 Hz, $NH_2CH_2CH_2$), 1.8 – 1.5 (4H, m, $NH_2CH_2CH_2CH_2$), 1.5 (9H, s, ^{t}*Bu*) ppm. **^{13}C NMR** (methanol-d_4, 101 MHz) δ = 170.5 , 164.2 , 83.5, 71.9, 57.2 , 32.1, 28.3 , 26.2 ppm. **FT-IR** ν_{max} (film) 3286, 2934, 1732, 1665, 1522, 1157, 1084 cm^{-1}. $[\alpha]_D^{25}$ = + 14.7° (c = 0.08 in MeOH). **HRMS** (ESI-TOF): calcd for $C_{11}H_{23}N_2O_4$ [M+H]$^+$ 247.1652, found 247.1664.

(2*S*,3*R*)-2-amino-2-carboxy-3-hydroxy-*N*,*N*,*N*-trimethylpentan-6-aminium (13)

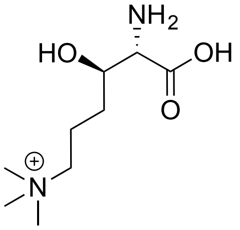

tert-Butyl (2*S*,3*R*)-6-amino-2-formamido-3-hydroxyhexanoate (**10**) (200 mg, 0.81 mmol) was dissolved in a 2:1 (v/v) MeCN/MeOH solution (8.1 ml). Methyl iodide (252 µl, 4.05 mmol) and *N*,*N*-diisopropylethylamine (183 µl, 1.05 mmol) were then added. The reaction mixture was stirred at room temperature until complete reaction was observed after ~4 h by TLC (5:4:1, $CHCl_3$/MeOH/AcOH). The reaction mixture was concentrated *in vacuo*, dissolved in a small amount of $CHCl_3$, then loaded onto a pre-equilibrated silica column (100% $CHCl_3$) and purified by flash column chromatography (0-100% MeOH (+10% formic acid) over ~10 column volumes). Ninhydrin active fractions were collected and concentrated. The residue was then dissolved in 6M $HCl_{(aq)}$ (8.1 ml) and stirred at room temperature overnight until complete reaction was observed by LC-MS. The reaction mixture was then concentrated to dryness. The resultant material was taken up in a minimal amount of H_2O, then purified using a cation exchange chromatography (Dowex® 1X8 200-400, Cl^- form) eluting in water. The ninhydrin active fractions were evaporated to dryness. The resultant residue was triturated several times with iPrOH to yield (**13**) a hygroscopic white powder (67 mg, 0.24 mmol, 30% over two steps); an e.r. of 3:1 was observed (Fig. S7A-B), as calculated by analysing the ^{1}H NMR spectra of the corresponding Mosher amides (Fig. S2).

^{1}H NMR (D_2O, 400 MHz) δ = 4.1 (1H, dt, *J*=9.0, 4.5 Hz, C*H*OH), 3.6 (1H, d, *J*=5.0 Hz, CH_2NH$_2$), 3.4 (2H, t, *J*=8.5 Hz, NH$_2$CH_2CH$_2$), 3.1 (9H, s, N(CH_3)), 2.1 – 1.8 (2H, m, NH$_2$CH$_2$CH_2CH_2), 1.8 – 1.5 (2H, m, NH$_2$CH$_2$CH_2CH_2) ppm. **^{13}C NMR** (D_2O, 101 MHz) δ = 173.1 , 69.7 , 60.0 , 53.5, 30.7, 19.8 ppm. **FT-IR** ν_{max} (film) 3363, 3026, 2919, 1735, 1622, 1489, 1222, 984 cm^{-1}. $[\alpha]_D^{25}$ = + 14.7° (c = 0.1 in H_2O/MeOH (1:1)). **HRMS** (ESI-TOF): calcd for $C_9H_{21}N_2O_3$ $[M]^+$ 205.1547, found 205.1547.

(2*S*,3*R*)-2,6-Diamino-3-hydroxyhexanoic acid di-hydrochloride salt (11)

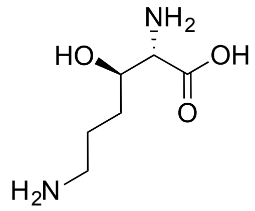

tert-Butyl (2*S*,3*R*)-3-(6-(dibenzylamino)propyl)-2,3-dihydrooxazole-2-carboxylate (**8**) (336 mg, 0.82 mmol) was dissolved in MeOH (6 ml) prior to addition of 5% (v/v) aqueous citric acid (2.3 ml). 10% palladium on carbon (100 mg/mmol) was then added, and the reaction vessel was put under a H_2 atmosphere using a balloon. The reaction mixture was stirred for 24 h (until complete conversion occurred, by LC-MS/TLC), then filtered through a short pad of Celite®, which was then further washed with MeOH. The filtrate was concentrated *in vacuo* and purified by flash column chromatography (CH_2Cl_2/MeOH, 100/0 to 30/70). Ninhydrin active fractions were collected and concentrated to give a clear oil, which was analysed by ^{1}H NMR. The oil was dissolved in 3M $HCl_{(aq)}$ and stirred overnight until complete reaction was observed by LC-MS and TLC. The reaction mixture was then concentrated to dryness to yield amine (**11**) as a white powder (di-hydrochloride salt, 176 mg, 0.75 mmol, 91%, Fig. S5A-B).

^{1}H NMR (400 MHz, D_2O) δ 4.16 (1H, dt, *J*=9.5, 3.5 Hz, C*H*OH), 3.91 (1H, d, *J*=4.5 Hz, C*H*NH_2), 3.04 (2H, t, *J*=7.5 Hz, C$H_2$$NH_2$), 1.94 – 1.55 (4H, m, $NH_2CH_2CH_2CH_2$) ppm. **^{13}C NMR** (101 MHz, D_2O) δ 170.99, 68.57, 58.11, 38.99, 30.02, 23.30 ppm.

Crude (**11**) was purified by cation exchange column chromatography using Dowex® 50WX8 200-400 (protonated form) resin that had been washed with H_2O. Crude (**11**) was then dissolved in a minimal amount of H_2O and loaded onto the column, followed by washing with at least 3 column volumes of water. The product was eluted with 3% (v/v) NH_4OH in MeOH to give purified (**11**) as a clear oil (Fig. S6A-B).

^{1}H NMR (400 MHz, D_2O) δ 4.01 – 3.90 (1H, m, C*H*OH), 3.55 (1H, d, *J*=5.0 Hz, C*H*NH_2), 2.96 (2H, t, *J*=7.0 Hz, C$H_2$$NH_2$), 1.85 – 1.46 (4H, m, $NH_2CH_2CH_2CH_2$) ppm. **^{13}C NMR** (101 MHz, D_2O) δ 172.62, 69.11, 59.32, 39.09, 30.30, 23.33 ppm. **FT-IR** ν_{max} (film) 3033, 2923, 1698 cm^{-1}. $[\alpha]_D^{25}$ = + 6.8 (c = 1.83 in MeOH) (Lit $[\alpha]_D^{25}$ = +16.9, c = 1.83 MeOH)[8]. **HRMS** (ESI-TOF): calcd for $C_6H_{15}O_3N_2$ $[M+H]^+$ 163.1077, found 163.1078.

Synthesis of Mosher's esters for stereochemical investigation of (9)

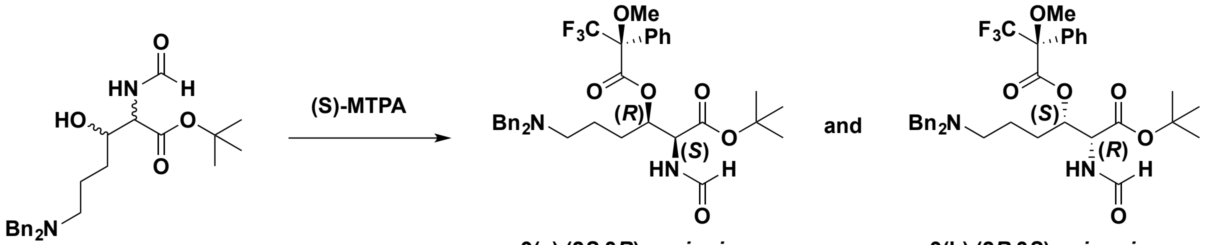

9(a) (2*S*,3*R*), *major isomer* 9(b) (2*R*,3*S*), *minor isomer*

Formamide (**9**) (1.0 eq., a mixture of stereoisomers) was dissolved in a solution of (*S*)-Mosher's acid (0.1 M, 1.5 eq) in CH_2Cl_2. A solution of *N,N'*-dicyclohexylcarbodiimide in CH_2Cl_2 (0.1 M, 2 eq.) was then added, followed by 4-dimethylaminopyridine (DMAP) in CH_2Cl_2 (0.1 M, 0.1 eq.). The resultant mixture was stirred at room temperature overnight to yield a precipitate that was subsequently filtered through a short pad of Celite®. The filtrate was then passed through a short pad of silica eluting with 100% EtOAc. After evaporating the filtrate to dryness, the residue was dissolved in $CDCl_3$ for analysis by ^1H NMR. The ^1H NMR spectra of the resultant Mosher esters are shown in Fig. S1A-C. The procedure was repeated with (*R*)-Mosher's acid to investigate the absolute stereochemistry of the C-3 position.

Synthesis of Mosher's amides of (13)

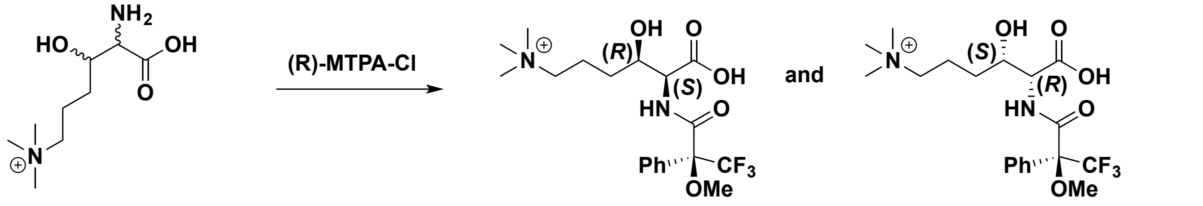

13(a) (2*S*,3*R*), *major isomer* 13(b) (2*R*,3*S*), *minor isomer*

Amine (**5**) (1.0 eq., a mixture of stereoisomers) was dissolved in a 10% $NaCO_3$ solution (0.2 M). (*R*)-Mosher's acid chloride solution in dioxane (0.2 M, 1.2 eq.) was then added to give a final reaction concentration of 0.1 M. This mixture was stirred for 3 h before being neutralized with a 1.0 M $HCl_{(aq)}$ solution and concentrated. The crude reaction mixture was concentrated to dryness and dissolved in D_2O for ^1H NMR analysis and e.r. determination; an e.r. of 3:1 was observed. The resultant ^1H NMR spectrum is shown in Fig. S2.

References

1 A. Kazaks, M. Makrecka-Kuka, J. Kuka, T. Voronkova, I. Akopjana, S. Grinberga, O. Pugovics and K. Tars, *Protein. Expr. Purif.*, 2014, **104**, 1-6.

2 M. Mantri, N. D. Loik, R. B. Hamed, T. D. Claridge, J. S. McCullagh and C. J. Schofield, *ChemBioChem*, 2011, **12**, 531-534.

3 T. R. Hoye, C. S. Jeffrey and F. Shao, *Nat. Protoc.*, 2007, **2**, 2451-2458.

4 G. R. Sullivan, J. A. Dale and H. S. Mosher, *J. Org. Chem.*, 1973, **38**, 2143-2147.

5 F. Sladojevich, A. Trabocchi, A. Guarna and D. J. Dixon, *J. Am. Chem. Soc.*, 2011, **133**, 1710-1713.

6 C. Cassani, R. Martin-Rapun, E. Arceo, F. Bravo and P. Melchiorre, *Nat. Protoc.*, 2013, **8**, 325-344.

7 L. Sidorowicz and J. Skarzewski, *Synthesis,* 2011, **5**, 708-710.

8 P. F. Hughes, S. H. Smith and J. T. Olson, *J. Org. Chem.*, 1994, **59**, 5799-5802.
